# Association between antiretroviral therapy and dental caries in children and adolescents with HIV: a systematic review and meta-analysis

**DOI:** 10.1186/s12903-025-06015-0

**Published:** 2025-05-10

**Authors:** Rubén Aguirre-Ipenza, Anthony Bautista-Pariona, Yolanda Viguria-Chavez, Alejandro Hector Huapaya-Cabrera, Franz Tito Coronel-Zubiate, Sara Antonieta Luján-Valencia, Elda Amaya-Riveros, Heber Isac Arbildo-Vega

**Affiliations:** 1 Faculty of Health Sciences, Universidad Continental, Lima, 15046 Perú; 2 Faculty of Human Medicine, Universidad Nacional del Santa, Ancash, Perú; 3 Instituto Privado de Estadística e Investigación en Salud, Lima, Perú; 4 School of Public Health, Universidad Peruana Cayetano Heredia, Lima, Perú; 5 Parasitology Unit, Alexander von Humboldt Institute of Tropical Medicine, Universidad Peruana Cayetano Heredia, Lima, Perú; 6 Faculty of Health Sciences, Universidad Privada del Norte, Lima, Perú; 7 Faculty of Health Sciences, Stomatology School, Universidad Nacional Toribio Rodríguez de Mendoza de Amazonas, Chachapoyas, Perú; 8 Faculty of Dentistry, Dentistry School, Universidad Católica de Santa María, Arequipa, Perú; 9 Department of Diagnostics and Surgery, School of dentistry, Paulista State University Julio de Mesquita Filho (UNESP), Araraquara, Sao Paulo, Brazil; 10 Institute of Government and Public Management, Universidad de San Martín de Porres, Lima, Perú; 11 Faculty of Dentistry, Dentistry School, Universidad de San Martín de Porres, Chiclayo, Perú; 12 Faculty of Human Medicine, Human Medicine School, Universidad de San Martín de Porres, Chiclayo, Perú

**Keywords:** Antiretroviral therapy, Dental caries, Children, HIV, Oral health, Systematic review

## Abstract

**Objective:**

To evaluate the evidence regarding the association between antiretroviral therapy and dental caries in children and adolescents with HIV.

**Methods:**

Searches were conducted in five international databases (PubMed, Scopus, EMBASE, CENTRAL, and LILACS) from the inception of records up to October 2024, including studies that examine the impact of antiretroviral therapy on caries in individuals under 18 years of age. The risk of bias was assessed using the Newcastle–Ottawa Scale. Quantitative synthesis was performed using the inverse variance method or Mantel–Haenszel method, depending on the type of outcome analyzed. Measures of association included odds ratios and mean differences, employing a random-effects model with a 95% confidence interval.

**Results:**

A total of 585 studies were identified, of which 17 were selected for qualitative review and 15 were included in the meta-analysis. The meta-analysis revealed a significantly higher risk of dental caries in children and adolescents with HIV undergoing antiretroviral therapy compared to those without the virus (odds ratio of 2.11; 95% CI: 1.41–3.17). Subgroup analysis showed a stronger association in case–control studies and for the DMFT index. The certainty of the evidence according to GRADE was rated as very low.

**Conclusion:**

Despite limited certainty, the results suggest that HIV under antiretroviral therapy is associated with a higher risk of dental caries. It is prudent to interpret these results with caution, considering the methodological limitations of the studies. However, given the possible relevance of this association for public health, it is recommended to consider specific dental care protocols for children and adolescents with HIV, as well as the need for preventive strategies integrated into HIV management.

**Supplementary Information:**

The online version contains supplementary material available at 10.1186/s12903-025-06015-0.

## Background

The human immunodeficiency virus (HIV) remains a significant global public health concern, especially in vulnerable populations such as children and adolescents. The World Health Organization (WHO) estimates that in 2023 there were approximately 39.9 million people living with HIV, with a high impact on vulnerable groups, including children [1]. Antiretroviral therapy (ART), previously classified as standard antiretroviral therapy (s-ART, consisting of one or two drugs) and highly active antiretroviral therapy (HAART, with three or more drugs from different classes), has transformed HIV management, significantly improving both the quality of life and life expectancy of infected patients [2, 3]. However, the prolonged use of these therapies can lead to side effects ranging from metabolic problems to alterations in the oral microbiota, a crucial aspect for dental and general health [4, 5].

In patients with HIV, the infection and continuous use of ART, including what was previously classified as HAART, can alter the balance of the oral microbiota due to their impact on the host's immune system and the bacterial equilibrium in the mouth. These changes can favor the growth of cariogenic bacteria, increasing the risk of developing dental caries [6].

Understanding how ART, in all its modalities, influences oral health is crucial to fully comprehend the side effects of the treatment [7]. Previous studies have suggested that children and adolescents with HIV may have a higher risk of developing dental caries due to alterations in the oral microbiota, an effect that could be exacerbated by ART [8, 9].

Exploring the relationship between ART and dental caries in children and adolescents with HIV is essential not only to improve clinical management strategies but also to develop effective preventive interventions [10]. Delving into this possible relationship would help dentists and physicians design specific dental care protocols for this population, which could potentially reduce the burden of dental caries and improve patients'quality of life.

Nevertheless, the direct impact of ART on dental caries in pediatric populations remains insufficiently documented. Although there are reviews that explore various aspects of oral health in patients with HIV [11, 12], most have not adopted structured approaches (such as Summary of Findings tables) to synthesize the quality of the evidence nor have they adequately addressed the influence of different subgroups. Consequently, a knowledge gap persists regarding the specific association between ART and the incidence of caries in children and adolescents. This gap underscores the need for a systematic review and meta-analysis to address these limitations. By focusing on the pediatric and adolescent population, this study aims to fill that gap through a detailed assessment of how ART, including previously utilized therapeutic regimens, influences the incidence and severity of dental caries.

The objective of this systematic review and meta-analysis is to evaluate the association between the administration of antiretroviral therapy and the incidence and severity of dental caries in children and adolescents with HIV. This study intends to provide solid and synthesized evidence that can guide clinical practices and health policies to improve the oral health of this vulnerable population.

## Methods

This systematic review follows the Preferred Reporting Items for Systematic Reviews and Meta-Analyses (PRISMA) guidelines [13], as detailed in Appendix 1. The protocol was registered in the International Prospective Register of Systematic Reviews – PROSPERO (CRD42024605937).

The PECO components of the question were as follows:

- P: Children and adolescents under 18 years
- E: Diagnosed with HIV who have received antiretroviral therapy
- C: Without HIV or diagnosed with HIV who have not received antiretroviral therapy
- O: Dental caries

To isolate the effect of ART on the incidence of caries in children and adolescents, we considered two types of comparator groups:

- HIV-negative individuals: This group serves to identify whether the combination of HIV infection plus ART exposure confers a higher risk of caries compared to those without infection.
- HIV-positive individuals without ART: By including children and adolescents with HIV who have not started antiretroviral therapy, we can specifically assess how treatment influences caries risk among individuals who share the same HIV status but differ in exposure to therapy.

### Eligibility criteria

Inclusion and exclusion criteria were established using the PECO method, focusing on comparative studies, including cohort, case–control, and cross-sectional studies that evaluated the association between antiretroviral therapy used in HIV treatment and dental caries in individuals under 18 years of age. This age range was chosen due to its importance in critical periods of dental development and general health. Studies specifically evaluating antiretroviral therapy as a factor associated with dental caries were included, presenting data on the presence or absence of caries, measured numerically (e.g., DMFT/dmft index) or categorically, in both exposed (HIV-infected individuals receiving antiretroviral therapy) and unexposed groups. No restrictions were applied regarding language, publication date, follow-up duration, or sample size. Review studies, conference abstracts, case reports, and studies conducted on animals or ex vivo samples were excluded.

### Search strategy

A systematic search was conducted for articles published up to October 2024 in Medline (via PubMed), Scopus, the Cochrane Library (CENTRAL), EMBASE, and LILACS, using search terms related to"dental caries,""antiretroviral therapy,"and"highly active antiretroviral therapy". Since our initial search in LILACS appeared too restrictive, we updated and expanded the search strategy in this database in February 2025 to ensure comprehensiveness. Additionally, gray literature was explored through OpenGrey and Google Scholar, reviewing the first 100 results on both platforms. References of included studies were also reviewed to identify potential additional studies not found in the databases. Finally, a manual search was performed in the leading journals specializing in HIV and general dentistry, including *Future Virology*, *BMJ Paediatrics Open*, *Indian Journal of Dental Research*, *Journal of Oral Pathology & Medicine*, and *BMC Oral Health*. The search strategy for each database is detailed in Table 1.

**Table 1:** Search strategy for each search engine

Database	Strategy		Results
PubMed	("antiretroviral therap*"[Title/Abstract] OR"antiretroviral therapy, highly active"[MeSH Terms] OR"HAART"[Title/Abstract] AND ("Dental Caries"[MeSH Terms] OR"Dental Caries Susceptibility"[MeSH Terms] OR"Root Caries"[MeSH Terms] OR ("dent*"[Title/Abstract] AND"cari*"[Title/Abstract]) OR"Decay"[Title/Abstract] OR"caries"[Title/Abstract] OR"tooth decay"[Title/Abstract] OR"carious lesion*"[Title/Abstract] OR"dental cavit*"[Title/Abstract] OR"carious dentin*"[Title/Abstract])		393
CENTRAL	#1	MeSH descriptor: [Antiretroviral Therapy, Highly Active] explode all trees	1630
	#2	(antiretroviral therap*):ti,ab,kw	9655
	#3	(HAART):ti,ab,kw	1322
	#4	MeSH descriptor: [Dental Caries] explode all trees	3727
	#5	MeSH descriptor: [Dental Caries Susceptibility] explode all trees	276
	#6	MeSH descriptor: [Root Caries] explode all trees	120
	#7	(dent* AND cari*):ti,ab,kw	9157
	#8	("decay"):ti,ab,kw	1791
	#9	("caries"):ti,ab,kw	9261
	#10	(tooth decay):ti,ab,kw	603
	#11	(carious lesion*):ti,ab,kw	1824
	#12	(dental cavit*):ti,ab,kw	4477
	#13	(carious dentin*):ti,ab,kw	1028
	#14	#1 OR #2 OR #3	9964
	#15	#4 OR #5 OR #6 OR #7 OR #8 OR #9 OR #10 OR #11 OR #12 OR #13	14322
	Final	#14 AND #15	80
Scopus	(INDEXTERMS("antiretroviral therapy, highly active") OR TITLE-ABS("antiretroviral therap*") OR TITLE-ABS(HAART)) AND (INDEXTERMS("Dental Caries") OR INDEXTERMS("Dental Caries Susceptibility") OR INDEXTERMS("Root Caries") OR (TITLE-ABS(dent*) AND TITLE-ABS(cari*)) OR TITLE-ABS(Decay) OR TITLE-ABS(caries) OR TITLE-ABS("tooth decay") OR TITLE-ABS("carious lesion*") OR TITLE-ABS("dental cavit*") OR TITLE-ABS("carious dentin*"))		396
Embase	('antiretroviral therapy, highly active'/exp OR'antiretroviral therap*':ti,ab OR HAART:ti,ab) AND ('Dental Caries'/exp OR'Dental Caries Susceptibility'/exp OR'Root Caries'/exp OR (dent*:ti,ab AND cari*:ti,ab) OR Decay:ti,ab OR caries:ti,ab OR'tooth decay':ti,ab OR'carious lesion*':ti,ab OR'dental cavit*':ti,ab OR'carious dentin*':ti,ab)		501
LILACS	(antiretroviral therapy, highly active) AND (dental caries)		4
LILACS (Expanded)	("Antiretroviral Therapy") OR ("Highly Active Antiretroviral Therapy") OR (HAART) OR ("antiretroviral therapy") OR ("antiretroviral treatment") AND ("Dental Caries") OR ("Dental Caries Susceptibility") OR ("Root Caries") OR (dent* AND cari*) OR ("caries") OR ("tooth decay") OR ("carious lesion") OR ("dental cavity") OR ("carious dentin") AND db:("LILACS") AND instance:"lilacsplus"		519

### Study selection

One author (RAI) gathered all references from the databases into the Rayyan QCRI web application (https://rayyan.qcri.org/) and removed duplicates. Before beginning the selection process, two authors (RAI and AHC) conducted a pilot test of the inclusion criteria to reduce errors in the selection process, using the first 100 studies. Subsequently, these authors independently reviewed the titles and abstracts of the retrieved publications to identify studies that potentially met the inclusion criteria. Conflicts regarding the inclusion of any publication were resolved through discussion with a third reviewer (EAR). All studies included based on titles and abstracts advanced to the full-text evaluation phase. These studies were independently assessed by the same review team members, and any discrepancies were resolved through discussion with a third reviewer (EAR).

### Data extraction

Four reviewers (ABP, YVC, FTCZ, SALV) were divided into two independent pairs. Each pair was assigned a portion of the included articles, from which they independently extracted the relevant data and entered them it into a Microsoft Excel spreadsheet. The extracted data included study characteristics (author, year of publication, country, study design, exposure measure, outcome), participant characteristics (age, gender, number of participants), and relative measures (crude and adjusted) with their confidence intervals obtained from the association between antiretroviral therapy in HIV patients and dental caries. Any disagreements were resolved through discussion or referral to a resolving reviewer (RAI).

### Study quality and certainty of evidence

Two reviewers (RAI and HIAV) independently assessed the risk of bias of included studies using the Newcastle Ottawa tool for case–control studies and cohort studies, and the adapted version for cross-sectional studies [14]. This tool consists of three domains: selection, comparability, and exposure or outcome. For case–control studies, the selection domain allows for up to 4 stars, comparability up to 2 stars, and exposure or outcome up to 3 stars. In the case of the adapted tool for cross-sectional studies, up to 5 stars can be assigned in the selection domain, 2 in comparability, and 3 in exposure or outcome. Higher scores indicate higher study quality. Stars are awarded based on the answer to each element's question. Responses are graded with one or two stars, signifying a lower risk of bias for the assessed component. In cases of disagreement, a third reviewer acted as an arbitrator (ABP).

### Data synthesis and analysis

The software Review Manager version 5.4.1 was used for the compilation and analysis of data from each study, using measures such as odds ratios and mean differences in random-effects models, providing 95% confidence intervals. Due to the observed heterogeneity among studies, random-effects models were applied, including inverse variance and Mantel–Haenszel methods for data analysis. To address heterogeneity beyond the I^2^ test and p-value, subgroup analyses were conducted based on study type, scale for measuring dental caries, comparator groups (HIV-negative or HIV-positive not yet receiving antiretroviral therapy), and type of therapy received (HAART versus standard ART). Additionally, heterogeneity was assessed using the I^2^ statistic, considering that heterogeneity may not be significant when I^2^ is below 40% [15]. Sensitivity analyses using fixed effects and other approaches were performed for studies with a risk of bias rating of 7 or more stars on the Newcastle–Ottawa Scale. It is noteworthy that a publication bias assessment was not performed due to the limited number of studies included in each analysis. The primary outcomes focused on the presence or absence of caries, measured numerically or categorically, in groups exposed and unexposed to antiretroviral therapy according to study design. Secondary outcomes included the mentioned subgroup analyses and proposed sensitivity analyses. In cases of missing data, and when necessary, we estimated the sample mean and standard deviation from sample size, median, interquartile range, or range, using the tool available at VassarStats (http://vassarstats.net). In situations where box plots were presented and means were reported without specifying the first or third quartile, we estimated these quartiles using AutoMeris (https://automeris.io), a computer vision-assisted software for extracting numerical data from data visualizations. Subsequently, with these data, we estimated the standard deviation.

### Certainty assessment

The authors, through consensus, categorized the certainty of evidence for all reported outcomes and were categorized as high, moderate, low, or very low following the criteria of the Grading of Recommendations, Assessment, Development, and Evaluations (GRADE) approach, using the GRADEpro software. The authors'consensus fully contextualized the rating of imprecision, risk of bias, inconsistency, and indirect evidence. We followed the GRADE guidance to communicate our findings and analyzed them using the GRADEpro GDT software (https://www.gradepro.org/).

## Results

### Search results

**Fig. 1:**
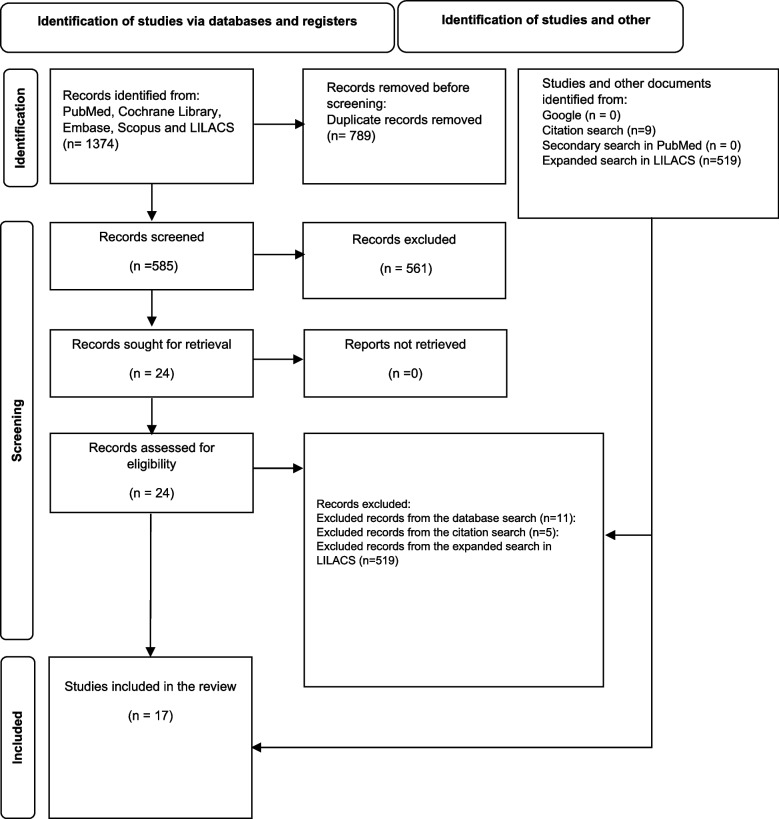
PRISMA flow diagram of the study selection process

We identified 1,374 records in the primary systematic search. After removing duplicates, 585 records were screened by title and abstract, of which 29 were reviewed in full text. In the expanded search in LILACS, 519 records were retrieved. However, none met the eligibility criteria. Finally, 17 met the inclusion criteria and were included in the analysis (Fig. 1). Excluded studies and reasons for their exclusion can be found in Table 2.

**Table 2:** Reason for exclusion of studies

Author(s)	Year	Reason for exclusion
Madigan et al. [16]	1996	No antiretroviral therapy reported
Costa et al. [17]	1998	No antiretroviral therapy reported
Tofsky et al. [18]	2000	No antiretroviral therapy reported
Filho et al. [19]	2009	Not the target population
Kelly et al. [20]	2009	Antiretroviral therapy not mentioned
Alves et al. [21]	2009	Antiretroviral therapy not mentioned
Nittayananta et al. [22]	2010	Not the target population
Liberali et al. [23]	2013	Not the target population
Rezaei et al. [24]	2014	Not the target population
Mandal et al. [25]	2016	Antiretroviral therapy not mentioned
Griffen et al. [26]	2019	Not the target population
Berrezouga et al. [27]	2024	Not the target population

### Characteristics of included studies

In total, nine cross-sectional studies [28–36], six case–control studies [37–42], and two cohort studies [9, 43] were included. In terms of origin, the articles were from different locations: Brazil [9, 37–41], India [29, 30, 33, 35, 42], Nigeria [32, 36, 43], Cambodia [34], Uganda [28], and West Africa, specifically Mali, Senegal, and Ivory Coast [31]. Regarding patient age, most studies included children and adolescents in a broad age range [9, 29–31, 33, 34, 38, 40, 42, 43]. Four studies focused on children under 6 years old [32, 37, 39]. Although the study by O'Connell et al. [36] did not focus on children under 6, it included participants with a mean age of 6.58 ± 1.92 years. Ferreira et al. [41] included youth over 14 years old, while Gainneos et al. [35] studied children between 9 and 12 years. Of the seventeen included studies, one was multicentric, conducted in different countries (Mali, Senegal, and Ivory Coast), while the other studies were conducted in a single country: Brazil, India, Nigeria, Cambodia, and Uganda.

### Caries data

Regarding caries data, all studies [9, 28–43] used validated World Health Organization (WHO) criteria; for example, DMFT/dmft (D: decayed teeth, M: teeth missing due to caries, F: filled teeth; in permanent and primary teeth, respectively).

### Type of antiretroviral therapy

Concerning the type of antiretroviral therapy, Cerqueira et al. [38], Coker et al. [32], Akhigbe et al. [43], Ferreira et al. [41], O'Connell et al. [36], Andrade et al. [40], and Ponnam et al. [29] report a high percentage of patients on HAART. Other studies, such as Kumar et al. [42], Rwenyonyi et al. [28], and Oliscovicz et al. [9], compare the effects of HAART with s-ART, presenting groups that allow evaluation of both therapies. Some studies, such as Gainneos et al. [35], Kikuchi et al. [34], Shrikanth et al. [30], Thejashwini et al. [33], De Jesus et al. [39], Castro et al. [37], and Rajonson et al. [31], lack specific details on the type of therapy used, limiting the accuracy of comparisons.

### HIV diagnosis

Regarding HIV diagnosis, most studies used standard serological tests such as ELISA and Western Blot to confirm infection [28, 29, 32, 34, 38, 40, 43]. Additionally, several studies used PCR tests for viral DNA detection regardless of age, although this method is particularly useful in children under 18 months, where maternal antibodies may interfere with serological test results [31, 32, 34, 36, 37]. Some studies followed specific criteria established by entities such as the CDC for HIV confirmation [37, 38, 43]. However, two studies did not specify the diagnostic methods used [30, 37].

### Data extraction for the meta-analysis

Regarding result extraction, nine studies reported data to calculate the crude OR, and ten studies reported their means and standard deviations. Therefore, for the meta-analysis, we considered studies where crude ORs were obtained and studies that reported the mean value of dental caries in the exposure groups with their respective standard deviations (Table 3).

**Table 3:** Characteristics of included studies

*Author(s)*	*Year*	*Country*	*Study type*	*Population*	*Number of participants (exposed or cases/controls)*	*Age*	Caries index	*Results* *OR (95% CI)*^b^ *MD (95% CI)*^c^ *CL (95% CI)*^d^	*Funding*
*Castro *et al*. *[37]	*2004*	Brazil	Case–control	*Children with HIV and HIV-negative*	*80 (40 VIH* + *and 40 VIH-)*	*2–5 years*	dmft/dmfs	*dmft: 1.52 (1.38–1.66)*^c^ *dmfs mean: G1: 7.85, G2: 3.43*	*Blackwell Munksgaard*
*Cerqueira *et al*. *[38]	*2010*	Brazil	Case–control	*Children with HIV and siblings without HIV*	*105 (65 VIH* + *and 40 VIH-)*	*2–13 years*	DMFT/dmft and number of cavitated carious teeth (CDT)	*DMFT: 0.50 (− 0.13 – 1.13)*^c^ *dmft: 2.20 (0.80–3.60)*^c^	*CNPq and FAPERJ*
*Rwenyonyi *et al*. *[28]	*2011*	Uganda	Cross-sectional	*Children with HIV*	*237 (118 with HAART and 119 without HAART)*	*1.5–12 years*	DMFT/deft	*DMFT: 2.06 (0.88–4.82)*^c^ *deft: 1.09 (0.65–1.82)*^c^	*Sida/SAREC*
*Ponnam *et al*. *[29]	*2012*	India	Cross-sectional	*Children with HIV and HIV-negative*	*285 (95 VIH* + *with HAART, 95 VIH* + *without HAART, and 95 VIH-)*	*5–15 years*	DMFT	*VIH* + *with HAART vs. VIH* + *sin HAART: 1.27 (0.69–2.35)*^b^* VIH* + *with HAART vs. VIH-: 0.91 (0.50–1.65)*^b^	*None*
*Shrikanth *et al*. *[30]	*2015*	India	Cross-sectional	*Children with HIV and HIV-negative*	*234 (78 VIH* + *and 156 VIH-)*	*3–17 years*	DMFT/dmft	*VIH* + *vs. VIH-: 3.77 (1.41–10.10)*^b^	*None*
*Oliscovicz *et al*. *[9]	*2015*	Brazil	Retrospective cohort	*Children with HIV*	*111 (3 groups: G1: 51 with HAART, G2: 46 with ART, and G3: 14 without medication)*	*2–16 years*	DMFT/deft	*Mean DMFT, G1: 1.9, G2: 1.6, G3: 3.0* *Mean deft, G1: 3.2, G2: 2.8, G3: 3.8*	*None*
*De Jesus *et al*. *[39]	*2017*	Brazil	Case–control	*Children with HIV and HIV-negative*	*280 (140 VIH* + *and 140 VIH-)*	*2–5 years*	dmft	*dmft: 2.46 (1.91–3.01)*^c^	*Not reported*
*Andrade *et al*. *[40]	*2016*	Brazil	Case–control	*Children with HIV and HIV-negative*	*99 (33 VIH* + *and 66 VIH-)*	*7–15 years*	DMFT/dmft and ICDAS	*VIH* + *vs. VIH-: 3.15 (0.98–10.15)*^b^ *DMFT: 1.34 (0.42–2.26)*^c^	*Not reported*
*Rajonson *et al*. *[31]	*2017*	Ivory Coast, Mali, Senegal	Cross-sectional	*Children with HIV and HIV-negative*	*838 (420 VIH* + *and 418 VIH-)*	*5–15 years*	DMFT/deft DMFS/defs	*VIH* + *vs. VIH-: 3.39 (2.41–4.76)*^b^* DMFT: 2.00 (1.93–2.07) ‡ deft: 2.00 (1.86–2.14)*^c^* DMFdefT: 2.00 (1.88–2.12)*^c^* DMFdefS: 5.00 (4.76–5.24)*^c^ *Probability of zero affected surfaces:* *Age* < *12 years:* *− 0.89 (− 1.53 to − 0.24)*^a,d^ *Age* ≥ *12 years:* *− 4.12 (− 7.43 to − 0.82)*^a,d^	*National Institutes of Health (NIH)*
*Coker *et al*. *[32]	*2018*	Nigeria	Cross-sectional	*Children with HI, HEU, HUU*	*335 (HI:100, HEU:105, HUU:130)*	*6–72 months*	dft/DMFT and NIDCR	*HI vs. HEU* + *HUU: 2.80 (1.35–5.81)*^b^ *HI vs. HEU: 4.10 (1.45–11.57)*^b^* HI vs. HUU: 2.22 (0.99–4.97)*^b^ *HI vs. HUU: 2.58 (1.04–6.40)*^b^ *HI vs. HEU: 2.22 (0.99–4.97)*^a^	*Fogarty AIDS International Training and Research Program, NIH*
*Thejashwini *et al*. *[33]	*2018*	India	Cross-sectional	*Children with HIV and general HIV-negative population*	*100 (50 VIH* + *and 50 VIH-)*	*5–18 years*	DMFT/dmft	*DMFT:0.84 (− 0.32 – 2.00)*^c^* dmft:− 0.64 (− 1.90 – 0.62)*^c^	*Not reported*
*Ferreira *et al*. *[41]	*2018*	Brazil	Case–control	*Children with HIV and HIV-negative*	*70 (34 VIH* + *and 36 VIH-)*	*14–24 years*	DMFT	*Not reported*	*CNPq,, CAPES, FAPERJ*
*Kikuchi *et al*. *[34]	*2021*	Cambodia	Cross-sectional	*Children with HIV and HIV-negative*	*482 (328 VIH* + *and 154 VIH-)*	*3–15 years*	DMFT/dmft	*DMFT: 0.70 (− 0.03 – 1.43)*^c^ *dmft: − 0.10 (− 1.18 – 0.98)*^c^ *DMFT: 1.85 (1.14–3.01)*^a^ *dmft: 0.65 (0.34–1.27)*^a^	*JSPS KAKENHI*
*Gainneos *et al*. *[35]	*2021*	India	Cross-sectional	*Children with HIV and HIV-negative*	*60 (30 VIH* + *and 30 VIH-)*	*9–12 years*	DMFT	*− 0.70 (− 1.36 – − 0.04)*^c^	*Self-funded*
*Akhigbe *et al*. *[43]	*2022*	Nigeria	Prospective cohort	*Children with HI, HEU, HUU*	*544 (HI:181, HEU:177, HUU:186)*	*4–11 years*	dmft/DMFT and ICDAS	*HI vs. HEU* + *HUU: 2.11 (1.41–3.17)*^b^* HI vs. HEU: 2.88 (1.71–4.84)*^b^* HI vs. HUU: 1.65 (1.04–2.61)*^b^ *HI vs. HEU* *Caries in any dentition: 1.58 (0.96–2.59)*^a^ *Caries in primary dentition: 1.41 (0.85–2.35)*^a^ *Caries in permanent dentition: 3.44 (1.25–4.49)*^a^	*National Institutes of Health-NIDCR R01DE028154*
*Kumar *et al*. *[42]	*2024*	India	Case–control	*Children with HIV*	*150 (95 HAART and 75 HAART-naïve)*	*6–18 years*	DMFT/dmft	*DMFT: 2.02 (1.24–2.80)*^c^ *dmft: 0.40 (− 0.38 –1.18)*^c^	*Not reported*
*O'Connell *et al*. *[36]	*2023*	Nigeria	Cross-sectional	*Children with HI, HEU, HUU*	*127 (HI: 49, HEU: 36, HUU: 42)*	*3–15 years*	DMFT/dmft and ICDAS	*HI vs. HEU* + *HUU: 2.14 (0.98–4.65)*^b^* HI vs. HEU: 3.46 (1.39–8.63)*^b^* HI vs. HUU 1.38 (0.56–3.41)*^b^	*National Institutes of Health (R01DE028154)*

^a^Adjusted Measures

^b^OR: Odds ratio

^c^MD: Mean Difference

^d^CL: logistic coefficient

G1: receiving HAART (highly active antiretroviral therapy, ≥ 3 drugs)

G2: receiving ART (antiretroviral therapy, ≤ 2 drugs)

G3: not receiving antiretroviral medication

NR: no record

### Risk of bias analysis of studies

**Fig. 2:**
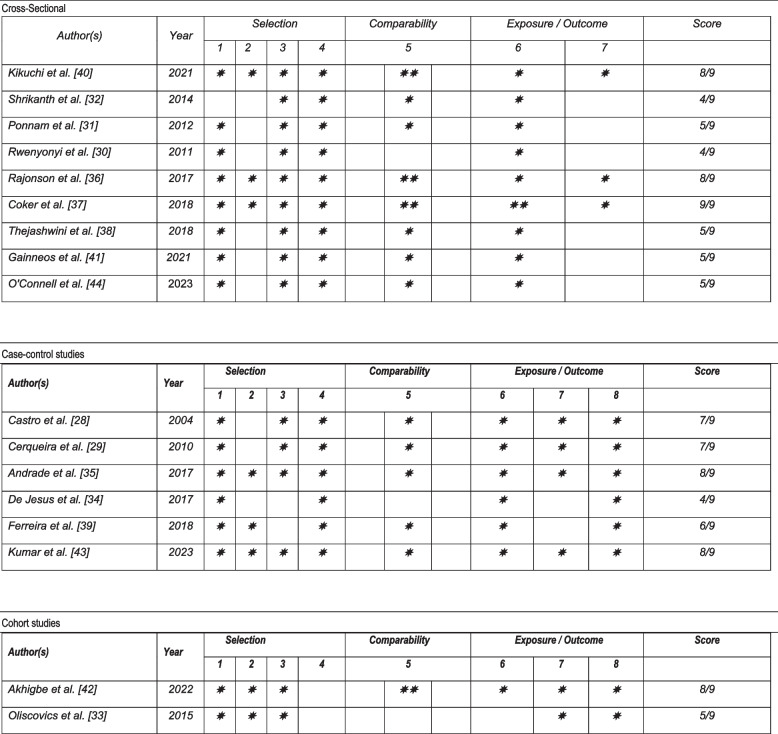
Risk of bias

**Fig. 3:**
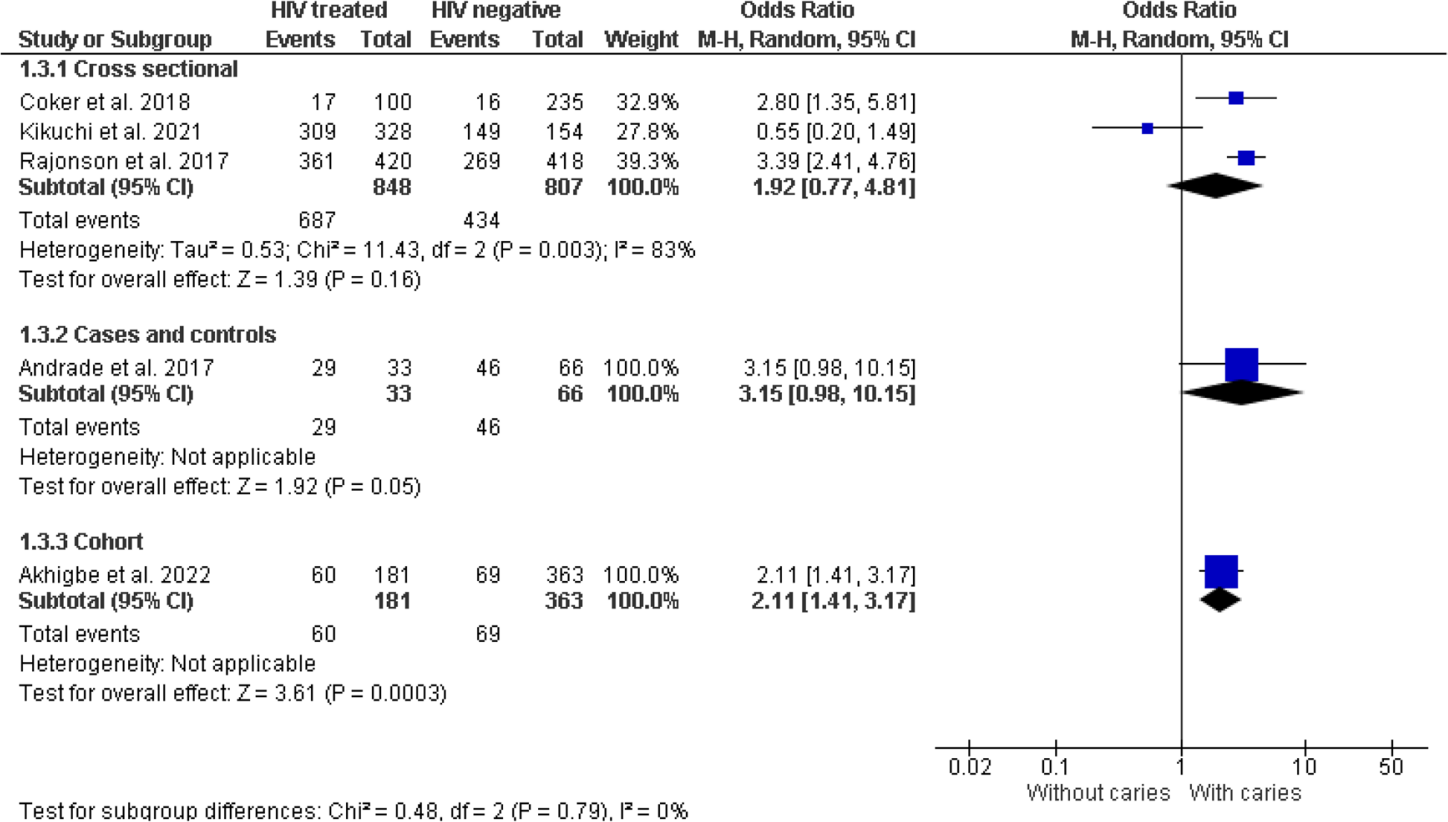
Forest plot of sensitivity analysis with studies scoring 7 or more stars on the Newcastle–Ottawa Scale for risk of bias assessment

A risk of bias analysis was conducted on six case–control studies and two cohort studies using the Newcastle–Ottawa Scale (NOS), and on nine cross-sectional studies using a modified version of the NOS for cross-sectional studies. The included cross-sectional studies presented a significant risk of bias, primarily in the Selection domain, specifically in the items “Sample size” and “Statistical test.” The case–control studies also showed a risk of bias in the Selection domain, especially in the “Sample size” item. The cohort studies similarly exhibited a risk of bias in the Selection domain, specifically in the “Demonstration that the outcome of interest was not present at the start of the study.” This situation hinders the appropriate extrapolation of results, which should be taken into account when interpreting the findings of this review (Fig. 2). Nevertheless, there were no differences in the direction of the effect between the overall findings and those obtained when evaluating only studies with a low risk of bias (Fig. 3).

### Quantitative analysis: results synthesis

**Fig. 4:**
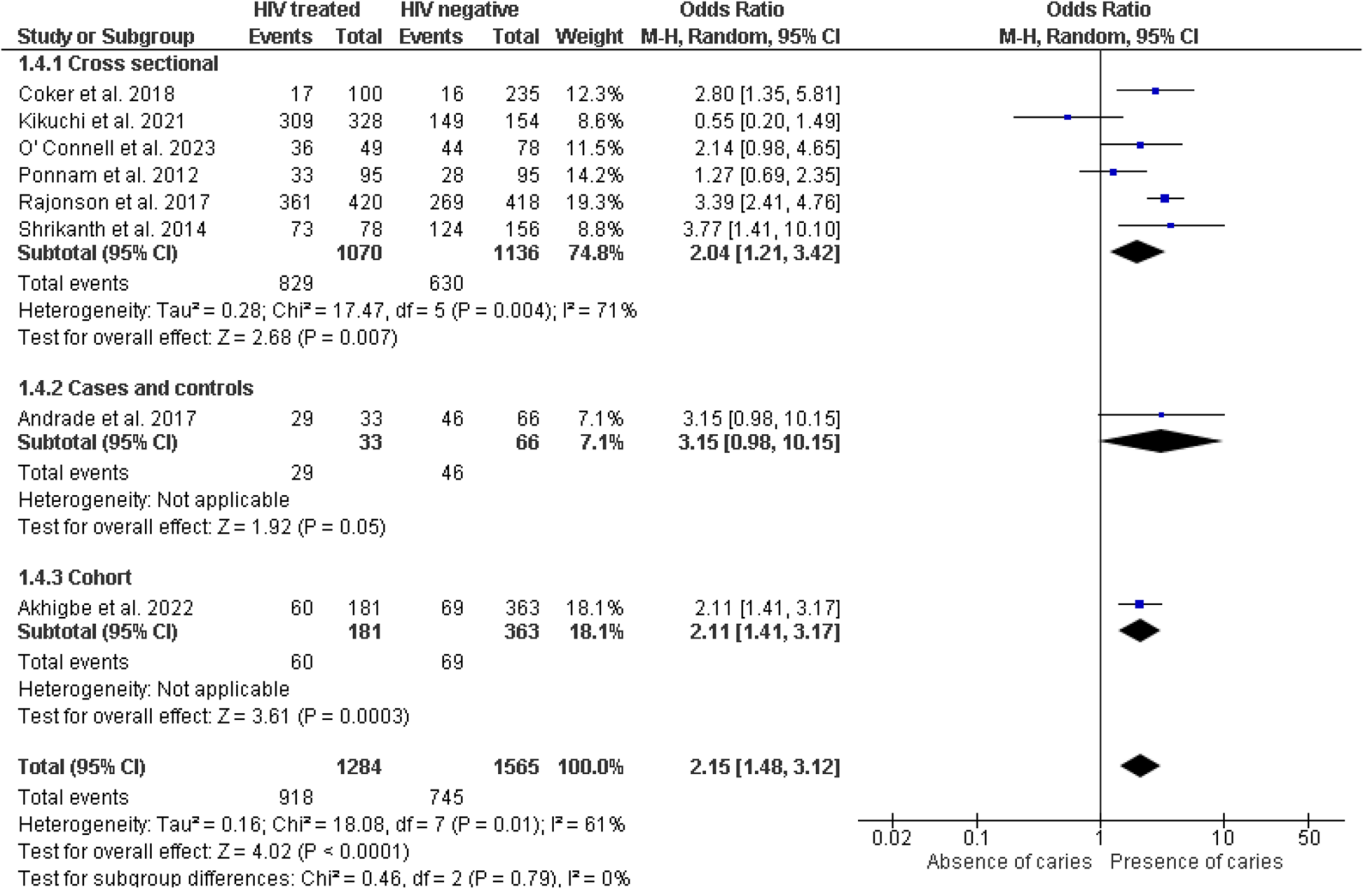
Forest plot comparing the presence of dental caries in individuals with HIV under antiretroviral therapy and individuals without HIV, according to study design

**Fig. 5:**
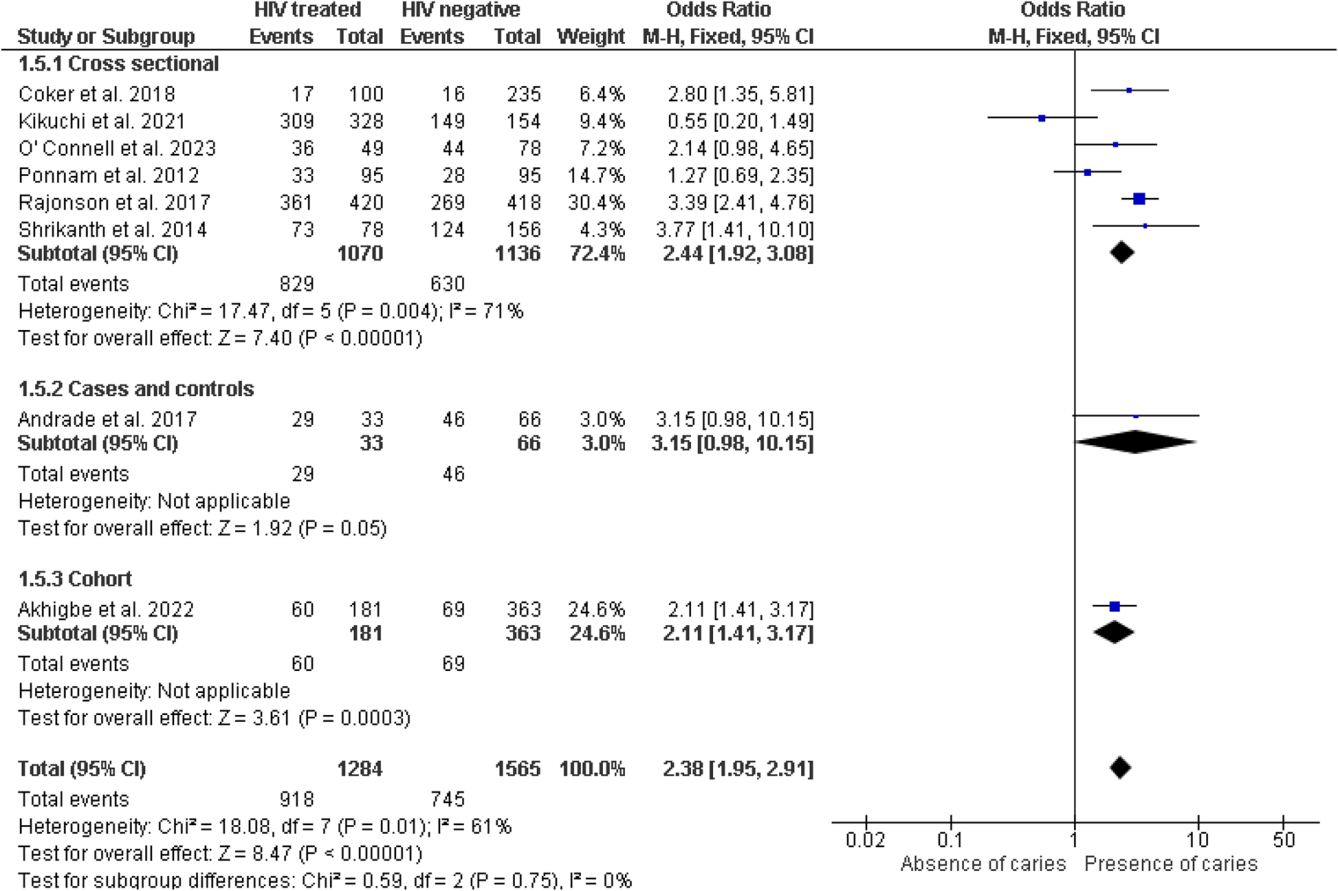
Forest plot of sensitivity analysis with fixed-effects model comparing dental caries in individuals with HIV under antiretroviral therapy and individuals without HIV, according to study design

Fifteen of the seventeen included studies [28–39, 41–43] provided data for the meta-analysis. The studies by Oliscovics et al. [9] and Ferreira et al. [40] were not included in the quantitative analysis. The first study was excluded because it only reported the mean value without dispersion measures such as standard deviation. The second study did not report numerical results. However, these studies reported conclusions indicating an association between HIV patients on ART and dental caries. The meta-analysis suggests significantly higher odds of dental caries in the HIV group receiving some form of antiretroviral treatment compared to the non-HIV group, in both cross-sectional studies (OR = 2.04, 95% CI 1.21–3.42, *p* = 0.007) and cohort study (OR = 2.11, 95% CI 1.41–3.17, *p* < 0.001). However, a case–control study reported no differences (OR = 3.15, 95% CI 0.98–10.15, *p* = 0.05) (Fig. 4). No statistically significant heterogeneity was detected between study designs (I^2^ = 0%, *p* = 0.79), and a similar result was obtained in the sensitivity analysis using a fixed-effects model (Fig. 5).

### Subgroup analysis

#### DMFT and dmft in HIV-positive patients (s-ART/HAART) vs. HIV-negative

**Fig. 6:**
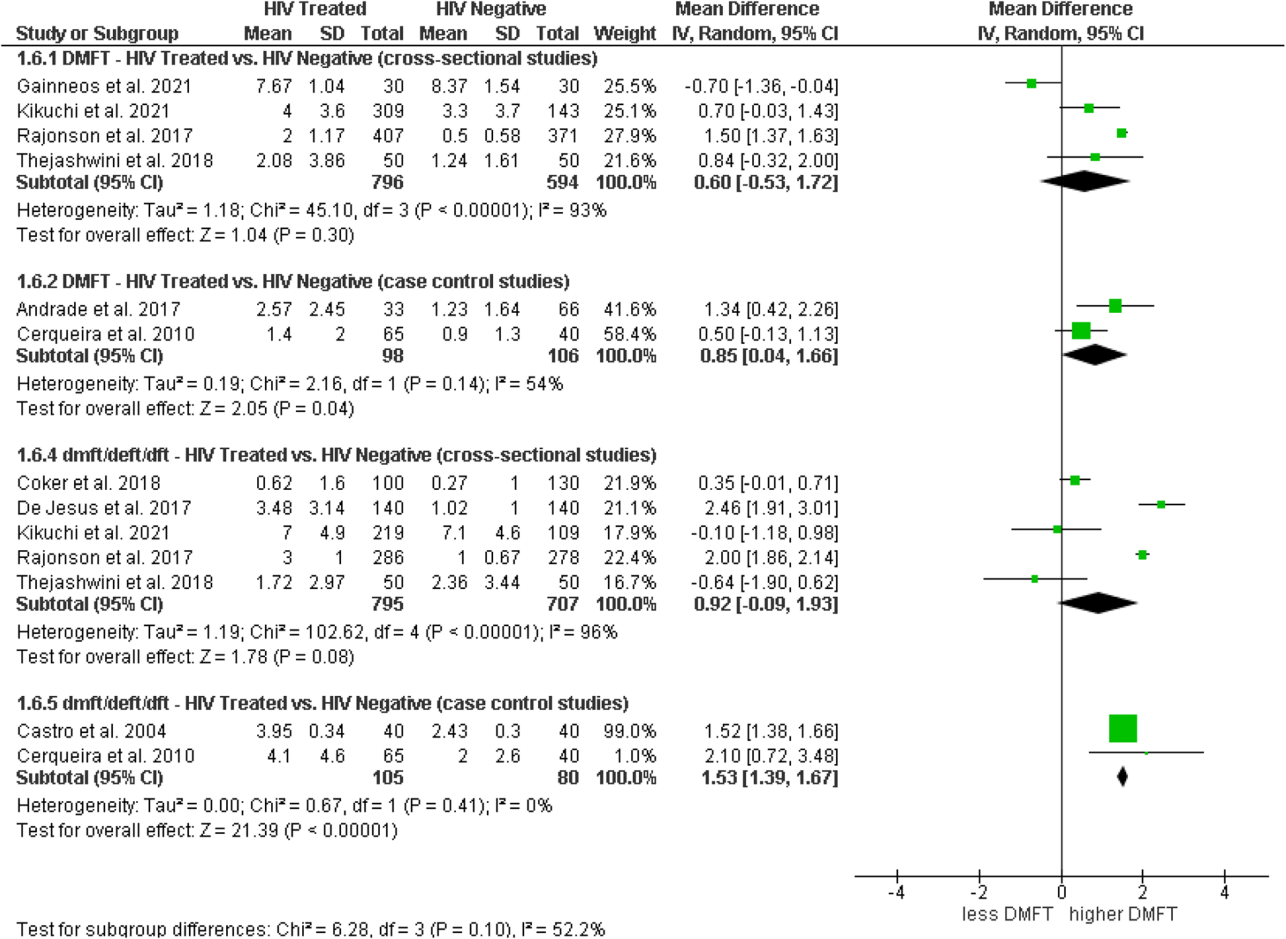
Forest plot of subgroup analysis comparing DMFT/dmft indices in individuals with HIV under treatment versus HIV-negative individuals, by study design

The subgroup analysis evaluated the caries indices (DMFT and dmft) in HIV-positive patients receiving antiretroviral therapy (s-ART/HAART) compared to HIV-negative individuals, differentiating the results by study design. For the DMFT index, cross-sectional studies (*n* = 4), with a total of 796 HIV-positive patients and 594 controls, revealed a mean difference (MD) of 0.60 (95% CI: − 0.53, 1.72), without statistical significance (*p* = 0.30) and with high heterogeneity (I^2^ = 93%). In case–control studies (*n* = 2), the MD was 0.85 (95% CI: 0.04, 1.66), showing significance (*p* = 0.04) and moderate heterogeneity (I^2^ = 54%), suggesting a possible association between HIV under ART and an increased DMFT index in this study type. In cross-sectional studies for the primary dentition index dmft/deft/dft, an MD of 0.92 (95% CI: − 0.09, 1.93) was observed, without significance (*p* = 0.08) but with high heterogeneity (I^2^ = 96%). Meanwhile, case–control studies showed a significant MD of 1.53 (95% CI: 1.39, 1.67) with no evidence of heterogeneity (I^2^ = 0%; *p* = 0.85), indicating that HIV under ART could be associated with a higher dmft index in this context (Fig. 6).

#### DMFS and dmfs in HIV-positive patients (s-ART/HAART) vs. HIV-negative

**Fig. 7:**
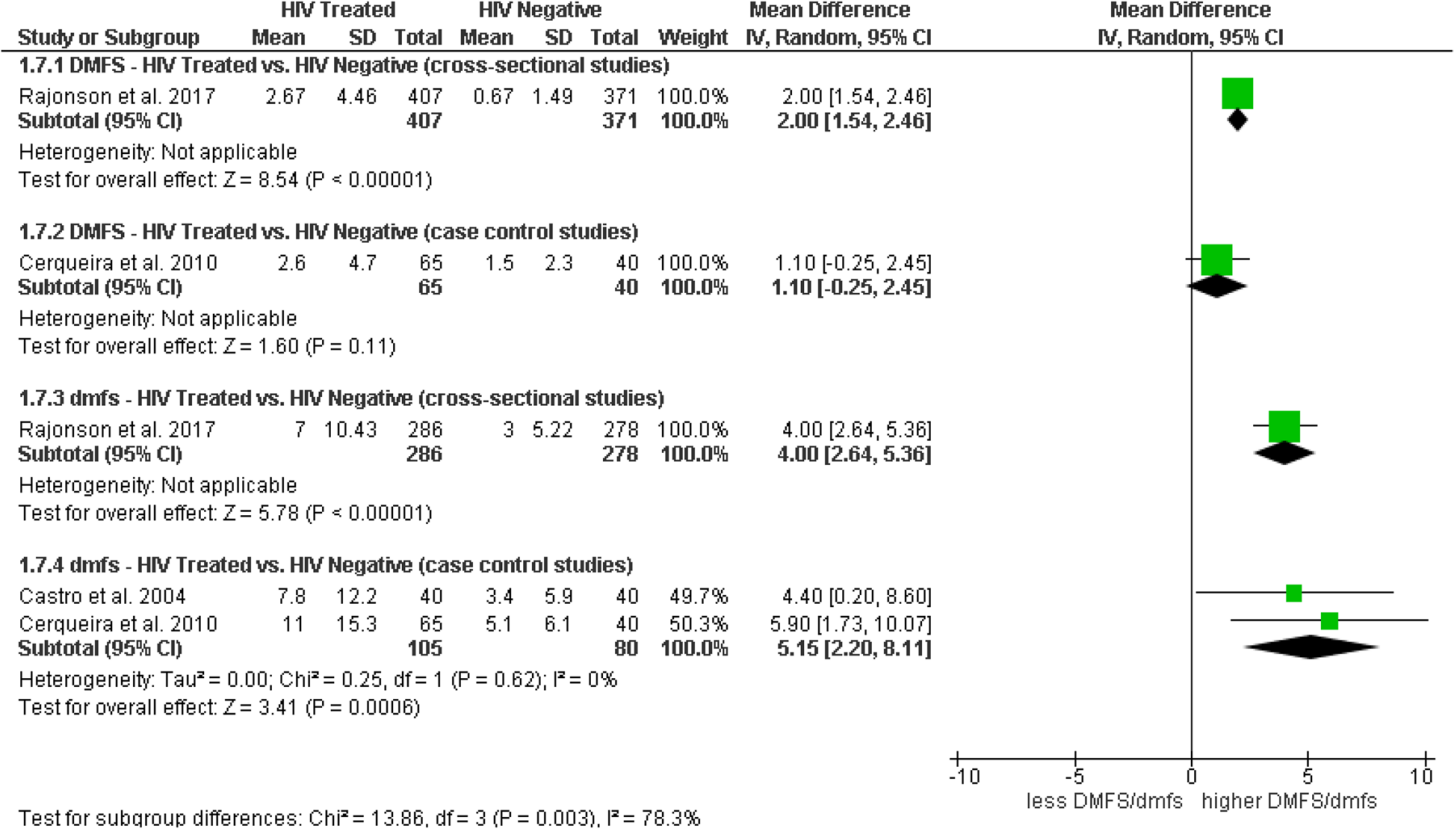
Forest plot of subgroup analysis comparing DMFS/dmfs indices in HIV-treated individuals versus HIV-negative individuals, by study design

An additional subgroup analysis evaluated differences in the DMFS and dmfs indices between HIV-positive individuals under ART and HIV-negative individuals. For the DMFS index, mixed results were observed: in cross-sectional studies, a significant mean difference of 2.00 (95% CI: 1.54, 2.46) was reported, indicating a higher caries index in the HIV-positive group; whereas, in case–control studies, the difference was not significant, with a mean of 1.10 (95% CI: − 0.25, 2.45). Regarding the dmfs index, cross-sectional studies showed a significant difference with a mean of 4.00 (95% CI: 2.64, 5.36), and case–control studies found significant differences with a combined mean of 5.15 (95% CI: 2.20, 8.11), also suggesting a higher prevalence of caries in the HIV-positive group compared to controls. The test for subgroup differences was significant (Chi^2^ = 13.86, df = 3, *p* = 0.003), indicating potential variations in the impact of ART depending on the evaluated index and study design (Fig. 7).

#### Impact of HAART on dental caries

**Fig. 8:**
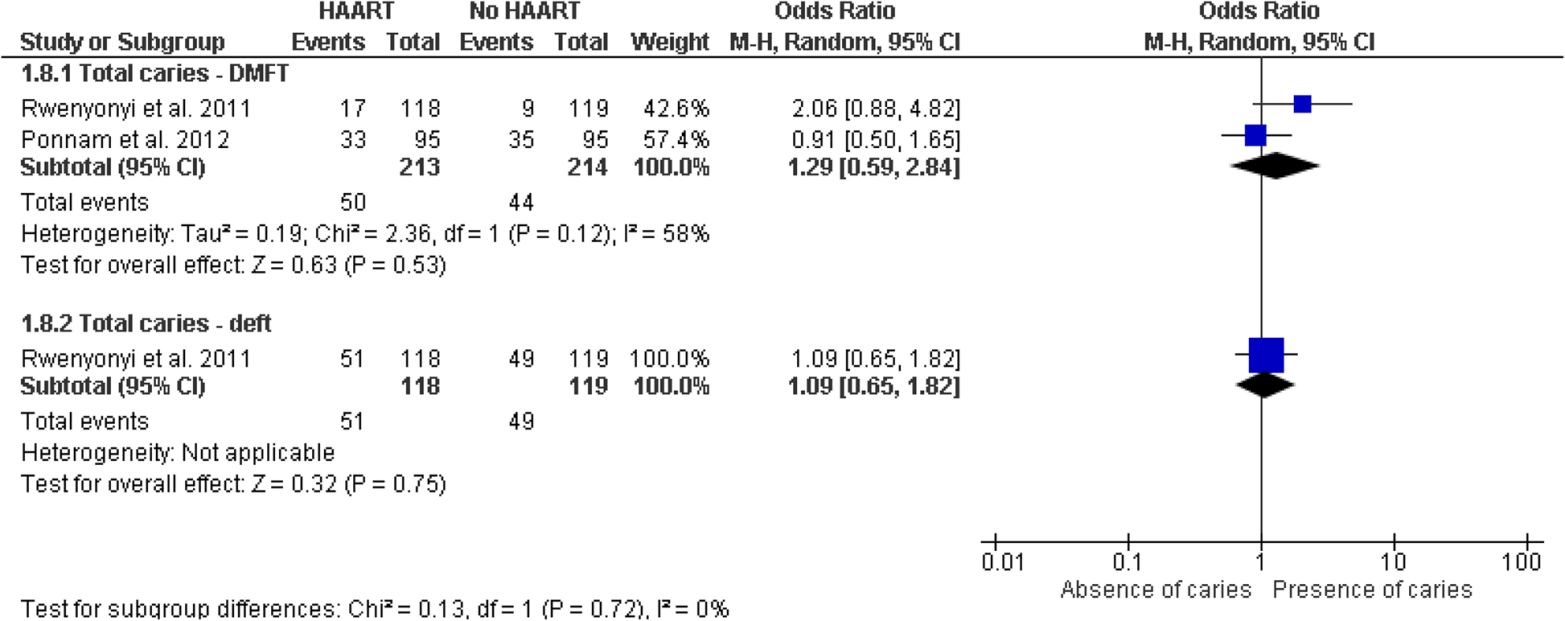
Forest plot of subgroup analysis comparing the probability of total caries (DMFT and deft indices) in HIV-positive individuals under HAART versus those not under HAART (cross-sectional studies)

**Fig. 9:**
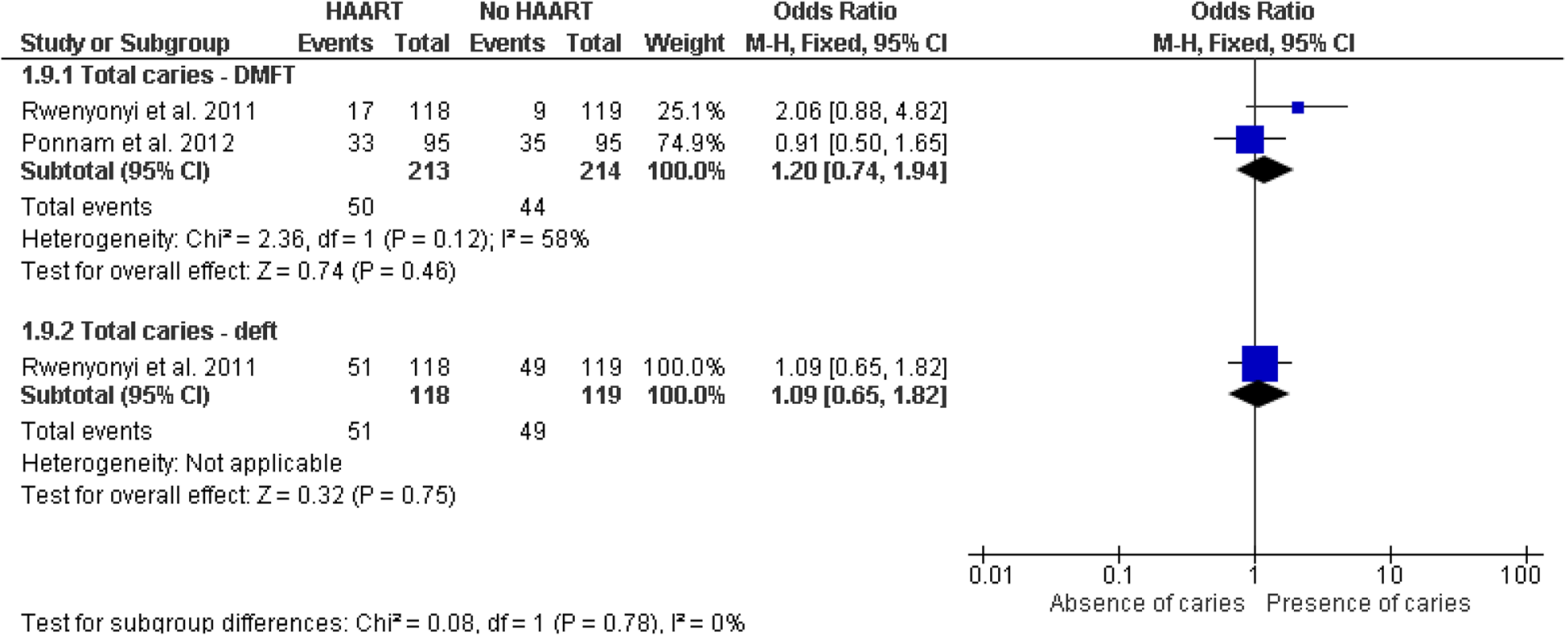
Forest plot of sensitivity analysis using a fixed-effects model comparing the probability of total caries (DMFT and deft indices) in HIV-positive individuals under HAART versus those not under HAART (cross-sectional studies)

**Fig. 10:**
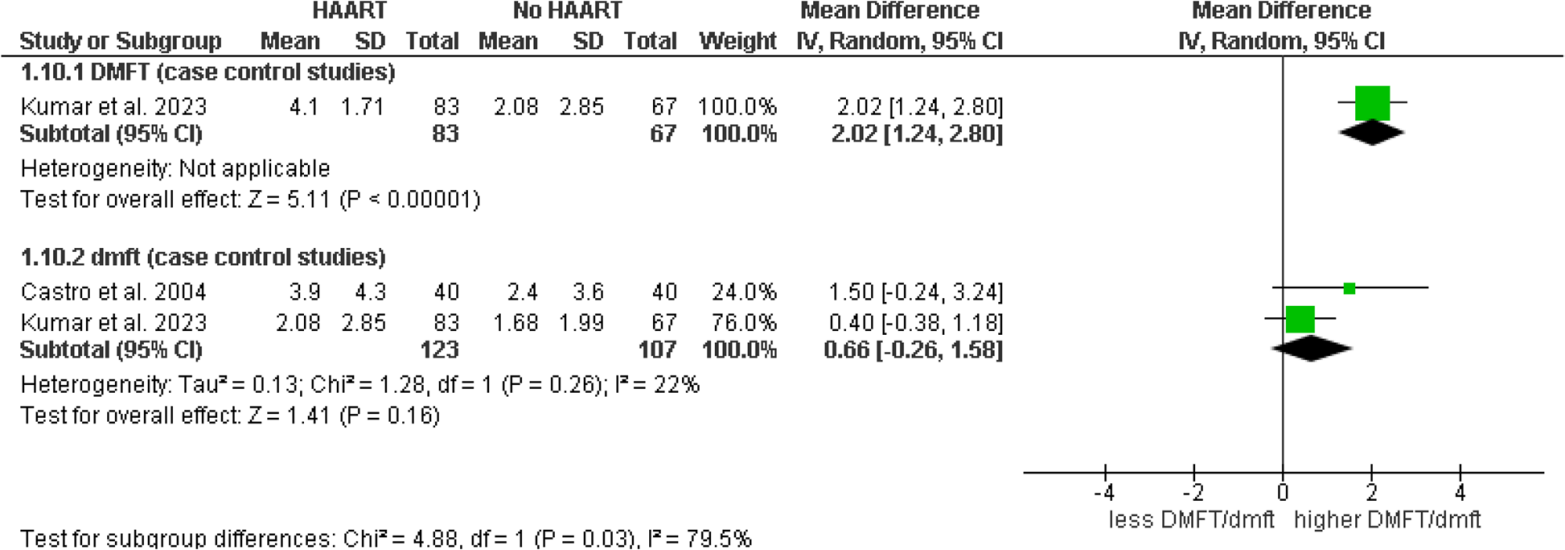
Forest plot of subgroup analysis comparing DMFT and dmft indices in HIV-positive individuals under HAART versus those not under (case–control studies)

In the subgroup analyses evaluating the impact of HAART on dental caries, a meta-analysis was first conducted on cross-sectional studies suggesting no significant differences in the probability of dental caries between the group of HIV-positive patients receiving HAART and those not receiving it. This was observed both in the total caries count with the DMFT index (OR = 1.29, 95% CI: 0.59–2.84, *p* = 0.53) and in the total count of carious teeth with the deft index (OR = 1.09, 95% CI: 0.65–1.82, *p* = 0.75) (Fig. 8). No statistically significant heterogeneity was detected among the caries indices used (I^2^ = 0%, *p* = 0.72), and the sensitivity analysis using a fixed-effects model showed consistent results (Fig. 9). Our primary analysis used a random-effects model, given the potential variability among different populations; however, a fixed-effects approach was used solely as a sensitivity analysis when heterogeneity was minimal (I^2^ = 0%) to confirm the consistency of our findings. Additionally, subgroups comparing the DMFT and dmft caries indices between HIV-positive patients under HAART and those without HAART were evaluated, yielding mixed results. In case–control studies for the DMFT index, a significant difference of 2.02 (95% CI: 1.24–2.80, *p* < 0.001) was reported, suggesting a higher DMFT index in the HAART group, while the analysis of the dmft index showed a non-significant mean difference of 0.66 (95% CI: − 0.26 – 1.58, *p* = 0.16) with low heterogeneity (I^2^ = 22%). The test for subgroup differences was significant (Chi^2^ = 4.88, *p* = 0.03, I^2^ = 79.5%), indicating potential variations in the impact of HAART depending on the evaluated index (Fig. 10). These results suggest a stronger association between HAART and the DMFT index, while the relationship with the dmft index is less conclusive, highlighting the need for additional studies and the importance of future research to confirm the observed relationship between antiretroviral therapy and caries indicators in HIV-positive patients.

#### Comparison with other control groups (HEU and HUU)

An additional analysis compared the presence of caries in HIV-infected children under antiretroviral therapy (HI) with different control groups: HIV-exposed but uninfected children (HEU) and unexposed and uninfected children (HUU). In cross-sectional and cohort studies, HIV-positive children showed a higher probability of caries compared to HEU (OR = 3.73, 95% CI: 1.88–7.40; and OR = 2.88, 95% CI: 1.71–4.84, respectively). Compared to HUU, cross-sectional and case–control studies showed mixed results, with a caries probability of OR = 1.80 (95% CI: 0.98–3.28) and OR = 1.61 (95% CI: 1.02–2.56), respectively (Suppl Mat. 1).

#### Certainty of evidence

We assessed the certainty of the evidence based on the main outcomes, divided by study design. For all outcomes, we identified a very low level of certainty, mainly due to concerns in the domains of risk of bias, heterogeneity, indirect evidence, and imprecision. Due to our very low confidence in the results, the evidence remains uncertain for all outcomes. However, our findings suggest that exposure to HIV and antiretroviral therapy could increase the incidence of dental caries by 163 additional cases (range: 46 to 255 more) per 1,000 individuals in cross-sectional studies and by 145 additional events (range: 68 to 233 more) per 1,000 individuals in a single cohort study. No differences were observed in a single case–control study. Furthermore, exposure to antiretroviral therapy could increase the DMFT index by 0.85 points (range: 0.04 to 1.66 more) and the dmft index by 1.53 points (range: 1.39 to 1.67 more) per 1,000 individuals in case–control studies. In cross-sectional studies, no significant differences were found in the DMFT and dmft indices (Table 4).

**Table 4:** Summary of finding

Outcomes	№ of participants (studies) Follow-up	Certainty of the evidence (GRADE)	Relative effect (95% CI)	Anticipated absolute effects*	
				Risk Without HIV	Risk difference with HIV under therapy
Dental caries (cross-sectional studies)	2206 (6 non-randomised studies)	⨁◯◯◯ Very low^a,b,c^	OR 2.04 (2.21 to 3.42)	555 per 1,000	163 more per 1,000 (46 more to 255 more)
Dental caries (case control studies)	33 cases 66 controls (1 non-randomised study)	⨁◯◯◯ Very low^d,e^	OR 3.15 (0.98 to 10.15)	697 per 1,000	182 more per 1,000 (4 fewer to 262 more)
Dental caries (cohort studies)	544 (1 non-randomised study)	⨁◯◯◯ Very low^e^	OR 2.15 (1.48 to 3.12)	190 per 1,000	145 more per 1,000 (68 more to 233 more)
Dental caries (cross-sectional studies) assessed with: DMFT index	1390 (4 non-randomised studies)	⨁◯◯◯ Very low^a,c,f,g^	-	-	MD 0.6 points higher (0.53 fewer to 1.72 higher)
Dental caries (case control studies) assessed with: DMFT index	204 (2 non-randomised studies)	⨁◯◯◯ Very low^c,h^	-	-	MD 0.85 points higher (0.04 higher to 1.66 higher)
Dental caries (cross-sectional studies) assessed with: dmft index	1502 (5 non-randomised studies)	⨁◯◯◯ Very low^c,g,i,j^	-	-	MD 0.92 points higher (0.09 fewer to 1.93 higher)
Dental caries (case control studies) assessed with: dmft index	185 (2 non-randomised studies)	⨁◯◯◯ Very low^,c,h^	-	-	MD 1.53 points higher (1.39 higher to 1.67 higher)

GRADE Working Group grades of evidence

High certainty: we are very confident that the true effect lies close to that of the estimate of the effect

Moderate certainty: we are moderately confident in the effect estimate: the true effect is likely to be close to the estimate of the effect, but there is a possibility that it is substantially different

Low certainty: our confidence in the effect estimate is limited: the true effect may be substantially different from the estimate of the effect

Very low certainty: we have very little confidence in the effect estimate: the true effect is likely to be substantially different from the estimate of effect

*CI* Confidence interval, *MD* Mean difference, *OR* Odds ratio

Explanations

^a^We downgraded the certainty of evidence by one level because half of the studies reported a higher risk of bias

^b^There is significant heterogeneity, confirmed by observing the forest plot and supported by the statistical value (I^2^ = 71%, *p* = 0.004)

^c^We downgraded by one level due to indirect evidence, as most of the studies partially align with the research question

^d^We downgraded by one level due to imprecision, considering that the study included a small sample size

^e^We downgraded by one level due to indirect evidence, as the study partially aligns with the research question

^f^There is significant heterogeneity, confirmed by observing the forest plot and supported by the statistical value (I^2^ = 93%, *p* = 0.00001)

^g^We downgraded by one level due to imprecision, as the pooled effect crossed the no-effect value

^h^There is significant heterogeneity, confirmed by observing the forest plot and supported by the statistical value (I^2^ = 54%, *p* = 0.14)

^i^We downgraded the certainty of evidence by one level because two of the three studies reported a higher risk of bias

^j^There is significant heterogeneity, confirmed by observing the forest plot and supported by the statistical value (I^2^ = 96%, *p* = 0.00001)

^k^We downgraded by one level due to indirect evidence, as the studies partially align with the research question

^l^We downgraded by one level due to imprecision, considering that the studies included small sample sizes

^*^The risk in the intervention group (and its 95% confidence interval) is based on the assumed risk in the comparison group and the relative effect of the intervention (and its 95% CI)

## Discussion

The main objective of this systematic review and meta-analysis was to evaluate the association between ART and dental caries in children and adolescents with HIV. Our findings revealed a significantly higher probability (OR 2.15) of dental caries in those under some form of antiretroviral therapy compared to individuals without HIV. This result reflects the diversity of findings reported in the studies included in the primary meta-analysis: four studies reported a higher risk of caries in children with HIV under antiretroviral therapy [30–32, 43], while another four did not find a significant association [29, 34, 36, 40].

The higher prevalence of dental caries in HIV patients under ART may be attributed to multiple factors. First, the systemic condition of HIV may reduce CD4 + T lymphocyte levels, leading to immunosuppression that increases the risk of colonization by cariogenic bacteria (6). Previous studies have reported an association between immunosuppression and greater severity of caries [37–40]. However, some authors, such as Shrikanth et al. [29], did not find a direct correlation between CD4 + count and dental caries, suggesting that other factors may contribute to this relationship.

Socioeconomic factors also play a crucial role. Souza et al. [17] reported that the frequency of caries was 21% higher in individuals with a family income equal to or lower than the minimum wage. This indicates that unfavorable economic conditions, commonly associated with HIV patients, may influence the prevalence of caries due to limitations in access to dental health services and oral hygiene education.

Furthermore, the immunological dysfunction associated with HIV may affect the concentration of salivary immunoglobulin A (IgA). Studies have found lower levels of salivary IgA in HIV-positive children, which could increase susceptibility to dental caries [25, 35]. However, it is important to consider that salivary IgA levels may also be affected by psychological factors such as stress and malnutrition [23, 35].

Antiretroviral therapy itself may influence oral health. Some studies suggest that ART may reduce salivary flow, a protective factor against caries [21]. Nevertheless, there is contradictory evidence, such as the study by Kikuchi et al. [34], which observed higher salivary flow in HIV-positive children. This suggests that the effect of ART on salivary flow may be modulated by other factors, such as dietary habits and diet.

Another aspect to consider is the high sugar content in pediatric formulations of antiretroviral medications. Syrups and suspensions with high sugar content can be fermented by oral bacteria, lowering intraoral pH and increasing the risk of caries [9].

This review also highlights the importance of regular follow-ups and preventive strategies in pediatric patients with HIV. Dental caries not only affects oral health but can also have systemic implications, impacting patients'quality of life and nutritional status [20].

Several studies have underscored the importance of adjusting for confounding variables when assessing the relationship between HIV infection, antiretroviral therapy, and dental caries. Coker et al. [32] found that HIV-infected children had a significantly higher risk of caries compared to uninfected children, with the association remaining significant after adjusting for sex, age, duration of breastfeeding, and membrane rupture (adjusted OR 2.58; 95% CI 1.04–6.40; *p* = 0.04). Similarly, Rajonson et al. [31] observed that the increased caries prevalence in HIV-positive children persisted in adjusted analyses accounting for factors like age, sex, sugary drink consumption, oral hygiene index, and tooth brushing frequency (logistic coefficient: –0.89; 95% CI –1.53 to –0.24; *p* = 0.007 for children under 12 years). Kikuchi et al. [34] reported that HIV-positive status was significantly associated with higher DMFT scores in permanent dentition even after adjusting for age, gender, socioeconomic status, BMI, and school functioning (adjusted OR 1.85; 95% CI 1.14–3.01; *p* < 0.05), while no significant association was found in deciduous dentition. Akhigbe et al. [43] also demonstrated that the higher prevalence of caries in HIV-infected children remained significant in adjusted analyses for permanent dentition (adjusted OR 3.44; 95% CI 1.25–9.49; *p* < 0.05) but not for deciduous dentition. These findings reinforce the results of our systematic review, highlighting that the association between HIV infection, antiretroviral therapy, and increased risk of dental caries in permanent teeth remains robust even after controlling for potential confounders.

The subgroup analysis in this meta-analysis shows that, in cross-sectional studies, there are no significant differences in the prevalence of caries between HIV-positive patients under HAART and those not receiving this therapy. This suggests that the influence of HAART on dental health is not determinant in studies of this design and highlights the importance of additional or contextual factors that could moderate this relationship.

The heterogeneity observed in this meta-analysis can be attributed to various factors, including differences in study designs (cross-sectional, case–control, and cohort), variability in caries diagnostic criteria (DMFT/dmft, ICDAS, etc.), the geographical and sociocultural diversity of the populations studied (Latin America, Africa, and Asia), and the wide age range (from early childhood to adolescence). In addition, some studies did not adequately control for socioeconomic factors or provide detailed data on the type, duration, and adherence to antiretroviral therapy, which makes it difficult to establish more homogeneous comparisons. To address this variability, random-effects models were used, and subgroup analyses were conducted, allowing for a more precise estimation of the overall association and exploration of potential sources of heterogeneity. Nevertheless, these methodological and contextual differences explain, at least in part, the variability in the results. Future research with standardized diagnostic criteria, tighter control of sociodemographic factors, and narrower age ranges could help reduce heterogeneity and provide greater clarity about the relationship between antiretroviral therapy and caries in children and adolescents with HIV.

When analyzed by study type, case–control studies show a potentially significant association between ART and an increase in the DMFT caries index, suggesting that this study design might better capture individual variations or factors associated with prolonged treatment. In contrast, cross-sectional studies present considerable heterogeneity, indicating that differences in the studied populations and methodology may influence the accuracy of the results.

The caries index for primary dentition (dmft) did not show statistically significant differences in cross-sectional studies; however, case–control studies revealed a more consistent association between ART and an increase in this index. This indicates that the relationship between HAART and caries may depend on both the study design and the specific indices used. Together, the findings highlight the complexity of the interaction between ART and dental health and the need to consider individual and design variations when interpreting the magnitude of this relationship.

The results of this systematic review and meta-analysis will benefit multiple groups, including pediatric patients with HIV, their families, health professionals, and public health policymakers. Although previous systematic reviews have explored various aspects of oral health in patients with HIV [11, 12], they have not adequately considered the influence of different subgroups, which can vary according to study design, type of treatment, caries index evaluated, or control group. Moreover, they have not incorporated structured approaches, such as Summary of Findings (SoF) tables, to synthesize the quality of the evidence. By addressing these gaps, our review provides a more comprehensive assessment of how ART influences the incidence and severity of dental caries in children and adolescents with HIV.

### Study limitations

Despite the rigorous methodology employed, our study presents some limitations. First, the inclusion of cross-sectional studies restricts the ability to establish causal relationships, as these designs only allow for the observation of associations at a specific point in time. Additionally, the included case–control studies may be subject to recall bias, which could compromise the validity of the associations.

Variability in the diagnostic criteria for dental caries (DMFT/dmft, ICDAS, among others), differences in antiretroviral therapy regimens, and inconsistencies in data reporting across studies may have contributed to the observed heterogeneity. Notably, many studies lacked detailed information on adherence to ART, treatment duration, and prior dental history, key factors that could influence caries development.

The risk of bias assessment revealed that several studies had limitations in representativeness and sample size, which may affect the generalizability of the results. Furthermore, confounding factors such as socioeconomic status could not be controlled in all studies, potentially influencing the observed association between ART and dental caries.

Finally, the absence of longitudinal studies prevents a deeper understanding of the temporal relationship between the initiation of ART and the development of dental caries. It is important to note that most of the included studies were conducted in specific geographic regions (Latin America, Africa, and Asia), where differences in healthcare infrastructure, nutritional status, and oral health policies may limit the applicability of our findings to other populations. Therefore, caution should be exercised when extrapolating these results to high-income settings, where factors such as access to dental services, cultural practices, and prevention policies may significantly influence this association. Conducting prospective studies is essential to determine whether ART is a causal factor in the increased incidence of dental caries.

### Recommendations

The relevance of this review lies in the evidence that HIV under antiretroviral therapy and dental caries could be interrelated. Therefore, we propose the following actions:

- Awareness and education: It is essential to raise awareness among healthcare professionals about the impact of ART on the oral health of HIV-positive children and adolescents. The implementation of specific educational programs can promote comprehensive care that considers both systemic and oral health.
- Preventive interventions and integration into health programs: The implementation of preventive and timely dental interventions is essential to prevent the progression of caries and facilitate its treatment in patients with HIV under antiretroviral therapy. Untreated caries can cause pain and affect quality of life, worsening the overall health status. Therefore, it is recommended to systematically integrate preventive care and dental interventions into national health programs for people with HIV, regardless of the establishment's category. In regions with limited human resources, it is suggested that a member of the healthcare team receive specific training from a dentist, whether a pediatric dentist or general dentist, for the early identification of oral health issues. Furthermore, it is imperative to reassess existing regulations to ensure an integral and effective approach to oral health care for this population, using the Technical Health Standard for Comprehensive Care of Children and Adolescents with HIV Infection in Peru [44] as a reference.
- Specific dental interventions: We recommend including specific dental interventions as part of national HIV prevention strategies. This involves not only oral evaluation but also the provision of necessary preventive and therapeutic treatments, such as fluoride applications, pit and fissure sealants, and sugar-free medication protocols where feasible.

## Conclusions

Our findings suggest a possible association between antiretroviral therapy and an increased likelihood of dental caries in children and adolescents with HIV. However, due to the observational nature of the included studies and the methodological limitations discussed, these results should be interpreted with caution. The heterogeneity in study designs, diagnostic criteria, and population characteristics highlights the need for more standardized and high-quality research in this field.

Longitudinal studies are required to establish a clearer understanding of the relationship between ART and dental caries, as well as to explore the potential underlying biological and behavioral mechanisms. Until more evidence is available, healthcare professionals should consider oral health as a relevant aspect of HIV management and promote preventive dental care for children and adolescents receiving ART.

## Supplementary Information

Supplementary Material 1

## Data Availability

The dataset supporting the conclusions of this article is included within the article. However, additional information can be requested from the corresponding author upon reasonable inquiry.

## Appendix

**Table:** PRISMA checklist

**Section and Topic **	**Item #**	**Checklist item **	**Location where item is reported**
**TITLE **			
Title	1	Identify the report as a systematic review.	First page 1
**ABSTRACT **			
Abstract	2	See the PRISMA 2020 for Abstracts checklist.	2, 3
**INTRODUCTION **			
Rationale	3	Describe the rationale for the review in the context of existing knowledge.	3, 4
Objectives	4	Provide an explicit statement of the objective(s) or question(s) the review addresses.	5
**METHODS **			
Eligibility criteria	5	Specify the inclusion and exclusion criteria for the review and how studies were grouped for the syntheses.	6
Information sources	6	Specify all databases, registers, websites, organisations, reference lists and other sources searched or consulted to identify studies. Specify the date when each source was last searched or consulted.	6, 7
Search strategy	7	Present the full search strategies for all databases, registers and websites, including any filters and limits used.	6, 7 (Table 1)
Selection process	8	Specify the methods used to decide whether a study met the inclusion criteria of the review, including how many reviewers screened each record and each report retrieved, whether they worked independently, and if applicable, details of automation tools used in the process.	8
Data collection process	9	Specify the methods used to collect data from reports, including how many reviewers collected data from each report, whether they worked independently, any processes for obtaining or confirming data from study investigators, and if applicable, details of automation tools used in the process.	9, 10
Data items	10a	List and define all outcomes for which data were sought. Specify whether all results that were compatible with each outcome domain in each study were sought (e.g. for all measures, time points, analyses), and if not, the methods used to decide which results to collect.	8
	10b	List and define all other variables for which data were sought (e.g. participant and intervention characteristics, funding sources). Describe any assumptions made about any missing or unclear information.	8
Study risk of bias assessment	11	Specify the methods used to assess risk of bias in the included studies, including details of the tool(s) used, how many reviewers assessed each study and whether they worked independently, and if applicable, details of automation tools used in the process.	8
Effect measures	12	Specify for each outcome the effect measure(s) (e.g. risk ratio, mean difference) used in the synthesis or presentation of results.	9, 10
Synthesis methods	13a	Describe the processes used to decide which studies were eligible for each synthesis (e.g. tabulating the study intervention characteristics and comparing against the planned groups for each synthesis (item #5)).	7
	13b	Describe any methods required to prepare the data for presentation or synthesis, such as handling of missing summary statistics, or data conversions.	8
	13c	Describe any methods used to tabulate or visually display results of individual studies and syntheses.	9, 10
	13 d	Describe any methods used to synthesize results and provide a rationale for the choice(s). If meta-analysis was performed, describe the model(s), method(s) to identify the presence and extent of statistical heterogeneity, and software package(s) used.	9, 10
	13e	Describe any methods used to explore possible causes of heterogeneity among study results (e.g. subgroup analysis, meta-regression).	9, 10
	13f	Describe any sensitivity analyses conducted to assess robustness of the synthesized results.	9, 10
Reporting bias assessment	14	Describe any methods used to assess risk of bias due to missing results in a synthesis (arising from reporting biases).	9, 10
Certainty assessment	15	Describe any methods used to assess certainty (or confidence) in the body of evidence for an outcome.	10
**RESULTS **			
Study selection	16a	Describe the results of the search and selection process, from the number of records identified in the search to the number of studies included in the review, ideally using a flow diagram.	10 (Fig.1)
	16b	Cite studies that might appear to meet the inclusion criteria, but which were excluded, and explain why they were excluded.	10 (Table 2)
Study characteristics	17	Cite each included study and present its characteristics.	11 (Table 3)
Risk of bias in studies	18	Present assessments of risk of bias for each included study.	13 (Fig. 2, Fig. 3)
Results of individual studies	19	For all outcomes, present, for each study: (a) summary statistics for each group (where appropriate) and (b) an effect estimate and its precision (e.g. confidence/credible interval), ideally using structured tables or plots.	13,14 (Fig. 4, Fig. 5)
Results of syntheses	20a	For each synthesis, briefly summarise the characteristics and risk of bias among contributing studies.	13 (Fig. 2, Fig. 3)
	20b	Present results of all statistical syntheses conducted. If meta-analysis was done, present for each the summary estimate and its precision (e.g. confidence/credible interval) and measures of statistical heterogeneity. If comparing groups, describe the direction of the effect.	14 (Fig. 5)
	20c	Present results of all investigations of possible causes of heterogeneity among study results.	14 (Fig. 5)
	20 d	Present results of all sensitivity analyses conducted to assess the robustness of the synthesized results.	14 (Fig. 5)
Reporting biases	21	Present assessments of risk of bias due to missing results (arising from reporting biases) for each synthesis assessed.	-
Certainty of evidence	22	Present assessments of certainty (or confidence) in the body of evidence for each outcome assessed.	17 (Table 4)
**DISCUSSION **			
Discussion	23a	Provide a general interpretation of the results in the context of other evidence.	18, 19, 20, 21, 22
	23b	Discuss any limitations of the evidence included in the review.	22, 23
	23c	Discuss any limitations of the review processes used.	22, 23
	23 d	Discuss implications of the results for practice, policy, and future research.	22
**OTHER INFORMATION**			
Registration and protocol	24a	Provide registration information for the review, including register name and registration number, or state that the review was not registered.	5
	24b	Indicate where the review protocol can be accessed, or state that a protocol was not prepared.	PROSPERO (CRD42024605937)
	24c	Describe and explain any amendments to information provided at registration or in the protocol.	-
Support	25	Describe sources of financial or non-financial support for the review, and the role of the funders or sponsors in the review.	26
Competing interests	26	Declare any competing interests of review authors.	26
Availability of data, code and other materials	27	Report which of the following are publicly available and where they can be found: template data collection forms; data extracted from included studies; data used for all analyses; analytic code; any other materials used in the review.	26

*From: * Page MJ, McKenzie JE, Bossuyt PM, Boutron I, Hoffmann TC, Mulrow CD, et al. The PRISMA 2020 statement: an updated guideline for reporting systematic reviews. BMJ 2021;372:n71. 10.1136/bmj.n71

## Abbreviations

HIV: Human Immunodeficiency Virus
ART: Antiretroviral Therapy
S-ART: Standard Antiretroviral Therapy
HAART: Highly Active Antiretroviral Therapy
PRISMA: Preferred Reporting Items for Systematic Reviews and Meta-Analyses
PROSPERO: International Prospective Register of Systematic Reviews
PECO: Population, Exposure, Comparator, Outcome
DMFT/dmft: Decayed, Missing, Filled Teeth (permanent and primary teeth)
WHO: World Health Organization
NOS: Newcastle Ottawa Scale
CI: Confidence Interval
OR: Odds Ratio
GRADE: Grading of Recommendations, Assessment, Development, and Evaluations
CDC: Centers for Disease Control and Prevention
ICDAS: International Caries Detection and Assessment System
PCR: Polymerase Chain Reaction
NIH: National Institutes of Health
NIDCR: National Institute of Dental and Craniofacial Research
ELISA: Enzyme-Linked Immunosorbent Assay

## References

[1] World Health Organization. HIV/AIDS Fact Sheet. Geneva: WHO; 2024. Available from: https://www.who.int/news-room/fact-sheets/detail/hiv-aids. Accessed 10 Oct 2024.

[2] UNAIDS. Global HIV & AIDS statistics—Fact sheet. Geneva: UNAIDS; 2024. Available from: https://www.unaids.org/en/resources/fact-sheet. Accessed 10 Oct 2024.

[3] Violari A, Cotton MF, Gibb DM, Babiker AG, Steyn J, Madhi SA, Jean-Philippe P, McIntyre JA; CHER Study Team. Early antiretroviral therapy and mortality among HIV-infected infants. N Engl J Med. 2008; 359(21):2233–44. 10.1056/NEJMoa0800971. 10.1056/NEJMoa0800971 PMC2950021 19020325

[4] Belkaid Y, Hand TW. Role of the microbiota in immunity and inflammation. Cell. 2014;157(1):121–41. 10.1016/j.cell.2014.03.011. 24679531 10.1016/j.cell.2014.03.011 PMC4056765

[5] Imahashi M, Ode H, Kobayashi A, Nemoto M, Matsuda M, Hashiba C, Hamano A, Nakata Y, Mori M, Seko K, et al. Impact of long-term antiretroviral therapy on gut and oral microbiotas in HIV-1-infected patients. Sci Rep. 2021;11(1):960. 10.1038/s41598-020-80247-8. 33441754 10.1038/s41598-020-80247-8 PMC7806981

[6] Beall CJ, Lilly EA, Granada C, Treas K, Dubois KR, Hashmi SB, Vazquez JA, Hagensee ME, Griffen AL, Leys EJ, et al. Independent Effects of HIV and Antiretroviral Therapy on the Oral Microbiome Identified by Multivariate Analyses. mBio. 2023;14(3):e0040923. 10.1128/mbio.00409-23. 10.1128/mbio.00409-23 PMC10294613 37071004

[7] Birungi N, Fadnes LT, Engebretsen IMS, Tumwine JK, Åstrøm AN; for ANRS 12174 AND 12341 study groups. Antiretroviral treatment and its impact on oral health outcomes in 5 to 7 year old Ugandan children: A 6 year follow-up visit from the ANRS 12174 randomized trial. Medicine (Baltimore). 2020;99(39):e22352. 10.1097/MD.0000000000022352. 10.1097/MD.0000000000022352 PMC7523782 32991450

[8] Mann AE, O'Connell LM, Osagie E, Akhigbe P, Obuekwe O, Omoigberale A, Kelly C, DOMHaIN Study Team, Coker MO, Richards VP. Impact of HIV on the Oral Microbiome of Children Living in Sub-Saharan Africa, Determined by Using an rpoC Gene Fragment Metataxonomic Approach. Microbiol Spectr. 2023;11(4):e0087123. 10.1128/spectrum.00871-23. 10.1128/spectrum.00871-23 PMC10434123 37428077

[9] Oliscovicz NF, Pomarico L, Castro GF, Souza IP. Effect of highly active antiretroviral therapy use on oral manifestations in pediatric patients infected with HIV. Indian J Dent Res. 2015;26(2):200–4. 10.4103/0970-9290.159169. 26096118 10.4103/0970-9290.159169

[10] Nakyonyi MG, Birungi N, Mwesigwa CL. Use of dental care services among adolescents living with HIV on antiretroviral treatment in Kampala, Uganda: a cross-sectional study. BMC Oral Health. 2024;24:654. 10.1186/s12903-024-04426-z. 38835044 10.1186/s12903-024-04426-z PMC11149271

[11] Lam PPY, Zhou N, Yiu CKY, Wong HM. Impact of Antiretroviral Therapy on Oral Health among Children Living with HIV: A Systematic Review and Meta-Analysis. Int J Environ Res Public Health. 2022;19(19):11943. 10.3390/ijerph191911943. 36231240 10.3390/ijerph191911943 PMC9565507

[12] Lam PPY, Zhou N, Wong HM, Yiu CKY. Oral Health Status of Children and Adolescents Living with HIV Undergoing Antiretroviral Therapy: A Systematic Review and Meta-Analysis. Int J Environ Res Public Health. 2022;19(19):12864. 10.3390/ijerph191912864. 36232165 10.3390/ijerph191912864 PMC9564723

[13] Page MJ, McKenzie JE, Bossuyt PM, Boutron I, Hoffmann TC, Mulrow CD, Shamseer L, Tetzlaff JM, Akl EA, Brennan SE, et al. The PRISMA 2020 statement: An updated guideline for reporting systematic reviews. Int J Surg. 2021;88:105906. 10.1016/j.ijsu.2021.105906. 33789826 10.1016/j.ijsu.2021.105906

[14] Modesti PA, Reboldi G, Cappuccio FP, Agyemang C, Remuzzi G, Rapi S, Perruolo E, Parati G; ESH Working Group on CV Risk in Low Resource Settings. Panethnic Differences in Blood Pressure in Europe: A Systematic Review and Meta-Analysis. PLoS One. 2016;11(1):e0147601. 10.1371/journal.pone.0147601. 10.1371/journal.pone.0147601 PMC4725677 26808317

[15] Higgins JPT, Thomas J, Chandler J, Cumpston M, Li T, Page MJ, Welch VA (editors). Cochrane Handbook for Systematic Reviews of Interventions, version 6.5 (updated August 2024). Cochrane, 2024. Accessed October 10^th^. Available from: https://training.cochrane.org/handbook/current. 10.1002/14651858.ED000142 PMC10284251 31643080

[16] Madigan A, Murray PA, Houpt M, Catalanotto F, Feuerman M. Caries experience and cariogenic markers in HIV-positive children and their siblings. Pediatr Dent. 1996;18(2):129–36. PMID: 8710715. Available from: https://www.aapd.org/globalassets/media/publications/archives/madigan-18-02.pdf. 8710715

[17] Costa LR, Villena RS, Sucasas PS, Birman EG. Oral findings in pediatric AIDS: a case control study in Brazilian children. ASDC J Dent Child. 1998;65(3):186–90. PMID: 9668947. Available from: https://pubmed.ncbi.nlm.nih.gov/9668947/. 9668947

[18] Tofsky N, Nelson EM, Lopez RN, Catalanotto FA, Fine DH, Katz RV. Dental caries in HIV-infected children versus household peers: two-year findings. Pediatr Dent. 2000;22(3):207–14. PMID: 10846731. Available from: https://pubmed.ncbi.nlm.nih.gov/10846731/. 10846731

[19] CavasinFilho JC, Giovani EM. Xerostomy, dental caries and periodontal disease in HIV+ patients. Braz J Infect Dis. 2009;13(1):13–7. 10.1590/s1413-86702009000100005. 19578624 10.1590/s1413-86702009000100005

[20] Kelly A, Soares LF, Pomarico L, Souza IPR. Risco e atividade de cárie em crianças com e sem infecção pelo HIV / caries risk and activity in HIV infected children and their controls. RGO (Porto Alegre).2009;57:217–222. Available from: https://pesquisa.bvsalud.org/gim/resource/ru/lil-522799.

[21] Alves CJ. Assessment of oral health status in pediatric patients infected with HIV virus: a case-control study [Master's thesis]. João Pessoa: Federal University of Paraíba; 2009. 94 p. Available from: https://repositorio.ufpb.br/jspui/handle/tede/6680.

[22] Nittayananta W, Talungchit S, Jaruratanasirikul S, Silpapojakul K, Chayakul P, Nilmanat A, Pruphetkaew N. Effects of long-term use of HAART on oral health status of HIV-infected subjects. J Oral Pathol Med. 2010;39(5):397–406. 10.1111/j.1600-0714.2009.00875.x. 20202089 10.1111/j.1600-0714.2009.00875.x PMC3217232

[23] Liberali SA, Coates EA, Freeman AD, Logan RM, Jamieson L, Mejia G. Oral conditions and their social impact among HIV dental patients, 18 years on. Aust Dent J. 2013;58(1):18–25. 10.1111/adj.12031. 23441788 10.1111/adj.12031

[24] Rezaei-Soufi L, Davoodi P, Abdolsamadi HR, Jazaeri M, Malekzadeh H. Dental caries prevalence in human immunodeficiency virus infected patients receiving highly active anti-retroviral therapy in Kermanshah, Iran. Cell J. 2014;16(1):73–8. Available from: https://pubmed.ncbi.nlm.nih.gov/24518976/. PMC3933441 24518976

[25] Mandal PK, Mitra M, Acharya S, Ghosh C, Mohanty S, Saha S. Salivary IgA versus HIV and Dental Caries. J Clin Diagn Res. 2016;10(9):ZC61-ZC64. 10.7860/JCDR/2016/19394.8531. 10.7860/JCDR/2016/19394.8531 PMC5072082 27790582

[26] Griffen AL, Thompson ZA, Beall CJ, Lilly EA, Granada C, Treas KD, DuBois KR 3rd, Hashmi SB, Mukherjee C, Gilliland AE, et al. Significant effect of HIV/HAART on oral microbiota using multivariate analysis. Sci Rep. 2019;9(1):19946. 10.1038/s41598-019-55703-9. 31882580 10.1038/s41598-019-55703-9 PMC6934577

[27] Berrezouga L, Kooli I, Marrakchi W, Neffati F, Najjar F, Chakroun M. Salivary biochemical parameters in people living with HIV on ART and dental caries: a cross-sectional study in Monastir, Tunisia. BMC Oral Health. 2024;24(1):35. 10.1186/s12903-023-03821-2. 38184520 10.1186/s12903-023-03821-2 PMC10771653

[28] Rwenyonyi CM, Kutesa A, Muwazi L, Okullo I, Kasangaki A, Kekitinwa A. Oral Manifestations in HIV/AIDS-Infected Children. Eur J Dent. 2011;5(3):291–8. Available from: https://pmc.ncbi.nlm.nih.gov/articles/PMC3137442/. PMC3137442 21769270

[29] Ponnam SR, Srivastava G, Theruru K. Oral manifestations of human immunodeficiency virus in children: An institutional study at highly active antiretroviral therapy centre in India. J Oral Maxillofac Pathol. 2012;16(2):195–202. 10.4103/0973-029X.98499. 22923890 10.4103/0973-029X.98499 PMC3424934

[30] Shrikanth M, Kumar A, Gangakhedkar R. Oral Health Status and Treatment Needs of Institutionalized HIV Positive and Non- HIV Positive Children and Adolescents between 3 To 17 Years of Age in Pune Maharashtra, India: A Comparative Study. Ind J Pre Clin Dent Res. 2015;3(1):92–98. Available from: http://www.ijocrweb.com/pdf/2015/January-March/9228_Review%20Paper.pdf.

[31] Rajonson N, Meless D, Ba B, Faye M, Diby JS, N'zore S, Datté S, Diecket L, N'Diaye C, Aka EA, et al. High prevalence of dental caries among HIV-infected children in West Africa compared to uninfected siblings. J Public Health Dent. 2017;77(3):234–243. 10.1111/jphd.12203. 10.1111/jphd.12203 28233316

[32] Coker M, El-Kamary SS, Enwonwu C, Blattner W, Langenberg P, Mongodin E, Akhigbe P, Obuekwe O, Omoigberale A, Charurat M. Perinatal HIV Infection and Exposure and Their Association With Dental Caries in Nigerian Children. Pediatr Infect Dis J. 2018;37(1):59–65. 10.1097/INF.0000000000001702. 28746260 10.1097/INF.0000000000001702 PMC5725234

[33] Thejashwini E, Vivek K, Sushmita D, Sreedevi A, Megha. A Study on Dental Caries Status among 5–18 Years HIV And Healthy Children-A Comparative Study. IOSR-JDMS. 2018;17(1):1–6. 10.9790/0853-1701130106.

[34] Kikuchi K, Yi S, Yasuoka J, Tuot S, Okawa S, Murayama M, Yem S, Chhoun P, Eng S, Huot C, Morokuma S. Oral health among HIV-positive and HIV-negative children in Phnom Penh, Cambodia: a cross-sectional study. BMJ Paediatr Open. 2021;5(1):e000992. 10.1136/bmjpo-2020-000992. 33782657 10.1136/bmjpo-2020-000992 PMC7957132

[35] Gainneos PD, Vasaviah SK, Duraisamy V, Krishnan R, John JB. A Comparative Evaluation of the Levels of Salivary IgA in HIV Affected Children and the Children of the General Population within the Age Group of 9–12 Years – A Cross-Sectional Study. Int J Cur Res Rev. 2021;13(5):61–5. 10.31782/IJCRR.2021.SP142.

[36] O’Connell LM, Mann AE, Osagie E, Akhigbe P, Blouin T, Soule A, Obuekwe O, Omoigberale A, Burne RA, Coker MO, et al. Supragingival mycobiome of HIV-exposed-but-uninfected children reflects a stronger correlation with caries-free-associated taxa compared to HIV-infected or uninfected children. Microbiol Spectr. 2023;11(6):e0149123. 10.1128/spectrum.01491-23. 37874172 10.1128/spectrum.01491-23 PMC10715047

[37] Castro GF, Souza IP, Lopes S, Stashenko P, Teles RP. Salivary IgA to cariogenic bacteria in HIV-positive children and its correlation with caries prevalence and levels of cariogenic microorganisms. Oral Microbiol Immunol. 2004;19(5):281–8. 10.1111/j.1399-302x.2004.00152.x. 15327638 10.1111/j.1399-302x.2004.00152.x

[38] Cerqueira DF, Portela MB, Pomarico L, de AraújoSoares RM, de Souza IP, Castro GF. Oral Candida colonization and its relation with predisposing factors in HIV-infected children and their uninfected siblings in Brazil: the era of highly active antiretroviral therapy. J Oral Pathol Med. 2010;39(2):188–94. 10.1111/j.1600-0714.2009.00857.x. 20040023 10.1111/j.1600-0714.2009.00857.x

[39] de Jesus MA, de Aguiar Ribeiro A, Lima PM, Chianca T, de Souza IP, de Araujo Castro GF. Influence of Caregiver's Sociodemographic Background on the Oral Health Status and Care of HIV-infected Children. J Dent Child (Chic). 2017;84(1):16–21. Available from: https://pubmed.ncbi.nlm.nih.gov/28387185/. 28387185

[40] Andrade NS, Pontes AS, Paz HES, de Moura MS, Moura LF, Lima MD. Molar incisor hypomineralization in HIV-infected children and adolescents. Spec Care Dentist. 2017;37(1):28–37. 10.1111/scd.12209. 27791275 10.1111/scd.12209

[41] Ferreira M, Cavalcanti E, Rubini N, Ferreira DC, Gonçalves LS, Colombo AP. Oral Status and Periodontal Microbiota of HIV-Infected Youth Infected By Vertical Transmission. Futur Virol. 2018;13(4):275–85. 10.2217/fvl-2018-0025.

[42] Kumar K, Subramaniam P, Prakash AJ. HAART medication and oral health status in children and adolescent HIV infected: A case control study. Spec Care Dentist. 2024;44(3):919–24. 10.1111/scd.12942. 37984407 10.1111/scd.12942

[43] Akhigbe P, Chukwumah NM, Folayan MO, Divaris K, Obuekwe O, Omoigberale A, Jedy-Agba E, Kim M, Charurat ME, Richards VP, et al. Age-specific associations with dental caries in HIV-infected, exposed but uninfected and HIV-unexposed uninfected children in Nigeria. BMC Oral Health. 2022;22(1):429. 10.1186/s12903-022-02421-w. 36167498 10.1186/s12903-022-02421-w PMC9512979

[44] Benites C, Vasquez R, Teran R, Loaiza K, Ruiz P, Huaman B, Morales M, Cardenas F, Romero S. Norma Tecnica de Salud para la Atencion Integral de las Ninas, Ninos y Adolescentes con Infeccion por el Virus de la Inmunodeficiencia Humana (VIH). Lima, Peru: MINSA; 2020. 79 pp. Available from: https://bvs.minsa.gob.pe/local/MINSA/5365.pdf.

