# Supplementary material for: Association between antiretroviral therapy and dental caries in children and adolescents with HIV: a systematic review and meta-analysis
Source: BMC Oral Health. 2025 May 10;25:700. doi: 10.1186/s12903-025-06015-0 (PMC12065305; doi:10.1186/s12903-025-06015-0)

**Suppl Mat. 1.** Forest plot of subgroup analysis comparing the probability of dental caries in HIV-positive treated children versus HEU (HIV-exposed uninfected) and HUU (HIV-unexposed uninfected) children, by study design.


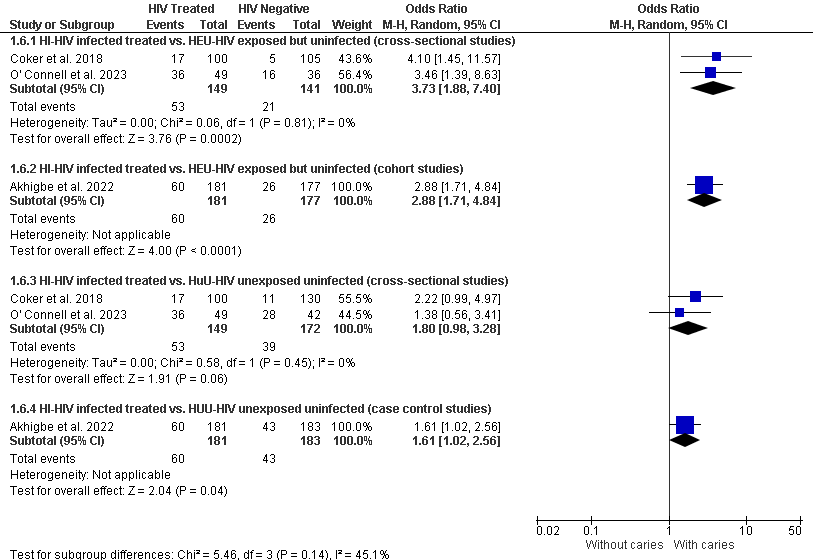

Supplement: Supplementary file 1 — Supplementary Material 1 [file 12903_2025_6015_MOESM1_ESM.docx]
